# Pilot first-in-human CCR2 PET/CT to detect abdominal aortic aneurysm wall instability

**DOI:** 10.7150/thno.108656

**Published:** 2025-04-13

**Authors:** Santiago Elizondo-Benedetto, Deborah Sultan, Ryan Wahidi, Mahdjoub Hamdi, Mohamed S. Zaghloul, Shahab Hafezi, Batool Arif, Laura K. McDonald, Kitty Harrison, Dakkota Thies, Gyu Seong Heo, Hannah Luehmann, Lisa Detering, J. Westley Ohman, Zachary J. Wanken, Luis A. Sanchez, Joseph E. Ippolito, Jie Zheng, Robert J. Gropler, Richard Laforest, Yongjian Liu, Mohamed A. Zayed

**Affiliations:** 1 Section of Vascular Surgery, Department of Surgery, Washington University School of Medicine, St. Louis, MO, USA.; 2 Department of Radiology, Washington University School of Medicine, St. Louis, MO, USA.; 3 Department of Biochemistry and Molecular Biophysics, Washington University School of Medicine, St. Louis, MO, USA.; 4 Division of Molecular Cell Biology, Washington University School of Medicine, St. Louis, MO, USA.; 5 Division of Surgical Sciences, Department of Surgery, Washington University School of Medicine, St. Louis, MO, USA.; 6 Department of Biomedical Engineering, McKelvey School of Engineering, Washington University School of Medicine, St. Louis, MO, USA.; 7 Veterans Affairs St. Louis Health Care System, St. Louis, MO, USA.

**Keywords:** AAA, CCR2, Molecular Imaging, RPI

## Abstract

**Objective:** In a pilot first-in-human study, we aimed to evaluate the feasibility of Positron Emission Tomography/Computed Tomography (PET/CT) imaging of C-C chemokine receptor type 2 (CCR2) to aid in the diagnosis of abdominal aortic aneurysm (AAA) instability.

**Rationale:** Risk stratification of AAAs is an unmet clinical need. Patients often remain asymptomatic until they acutely rupture. Current imaging techniques focus on AAA diameter and growth rate, neglecting key cellular and molecular processes.

**Methods:** A pilot, prospective, single-center, case-control study evaluated patients with and without AAAs. The study subjects received intravenous administration of a CCR2-specific radiotracer, followed by PET/CT assessment. Surgical AAA specimens were collected to evaluate CCR2 content and extracellular matrix integrity. PET/CT signals were evaluated in the AAA wall in the para-renal, mid-infrarenal, and aneurysm sac, and analyzed relative to patient demographics, AAA anatomical segmentation, and wall rupture potential index (RPI).

**Results:** The AAA group was elderly (70.7 ± 7.3), with an aneurysm diameter of 4.86 ± 0.75 cm, and a higher prevalence of hyperlipidemia and statin use. Regardless of the anatomical segment analyzed, AAA surgical patients demonstrated a higher CCR2 radiotracer signal in the aortic tissue than others. However, no correlation was observed between the radiotracer signal and the AAA diameter. Patients with a higher radiotracer signal, particularly in the AAA posterior wall of the maximum-diameter region, were significantly correlated with RPI (P = 0.03). Histomorphic analysis demonstrated significantly elevated CCR2 levels, along with increased macrophage infiltration, matrix metalloproteinase activity, and severe elastin degradation.

**Conclusions:** This first-in-human study demonstrated that CCR2 PET/CT molecular imaging is feasible and can identify increased wall instability in individuals with AAAs, especially in those at higher risk of disease progression.

## Introduction

Abdominal aortic aneurysm (AAA) is a life-threatening degenerative vascular disease characterized by transmural aortic macrophage infiltration, elastin degradation, and loss of vascular smooth muscle cells 1,2. AAAs typically remain asymptomatic until they rupture, leading to high mortality and a substantial burden on the healthcare system 3,4. AAA development has been associated with various risk factors, including smoking, hypertension, aging, and male sex 5. Nevertheless, the molecular mechanisms that lead to aneurysm initiation, progression, and rupture remain elusive 6,7.

Currently, AAA management lacks targeted medical therapies tailored specifically for this disease. Reliable imaging protocols are required to accurately assess the individual characteristics of each patient with an aneurysm, which can vary significantly. To date, AAA management largely centers on the measurement of aortic diameter, which serves as a limited marker for predicting the risk of rupture 8-13. Patients who do not meet the anatomical criteria for surgical repair are typically managed expectantly, even though a significant proportion of ruptured AAAs have diameters smaller than 5.5 cm (the typical diameter threshold for surgical intervention), and approximately 40% of patients with AAA diameters ranging from 7.0-10.0 cm do not experience rupture 11,13. This highlights the limited sensitivity of current anatomy-based technologies for identifying individuals who are more prone to AAA rupture 14. Alternative approaches such as the AAA rupture potential index (RPI) have been proposed 15. However, these mechanical stress measurement techniques lack crucial biomolecular characteristics that are necessary for patient-specific evaluations and do not address the persistent need for personalized care in the context of AAA disease.

The C-C chemokine receptor type 2 (CCR2) plays a crucial role in mediating pro-inflammatory monocyte trafficking to sites of inflammation in the arterial wall following injury 16. In preclinical studies, we demonstrated that CCR2 plays an important role in AAA formation and rupture 17-19. Genetic knockdown and pharmacological inhibition of CCR2 significantly reduced the production of matrix metalloproteinases (MMPs) and decreased the risk of AAA rupture 16-18. These findings suggest that using CCR2 as a theranostic biomarker may be useful for monitoring AAA progression. Consistent with this we previously observed that [^64^Cu]-DOTA-ECL1i, a radiotracer that selectively binds to CCR2, specifically accumulates in AAA tissue, and is predictive of AAA rupture in preclinical animal models 18,19. We therefore hypothesized that positron emission tomography/computed tomography (PET/CT) imaging of CCR2 can be used to assess disease severity in AAA tissue and may indicate anatomical regions with higher mechanical wall stress and instability.

## Methods

### Study groups

Consecutive male and female patients aged ≥30 years (with or without a history of smoking tobacco) with AAAs confirmed by a computed tomographic angiogram (CTA), as well as healthy volunteers, with no AAAs determined by screening ultrasound, were recruited in a pilot, prospective, single-center, case-control study (NCT04586452). AAA patients were selected for operative repair (surgical AAA; n = 5) or expectant medical management (non-surgical AAA; n = 5) as determined by the clinical care team and by using traditional aneurysm diameter on CTA (men ≥5.5 cm, women ≥5.0 cm). Healthy volunteers without AAAs (non-AAA controls, n = 9) were also included. The eligibility criteria and detailed protocol specifics are available in Supplementary Material.

### PET/CT imaging and analysis

All PET/CT studies were analyzed using the systematic protocol described in Supplementary Material. Static PET/CT and CTA were performed. Blood pool subtraction and aortic anatomical segmentation were also conducted (**Figure S1 & S2, Supplementary Material**). CCR2 radiotracer dosimetry and tissue-distribution assessment was performed as previously described 20. The complete statistical analysis is provided in Supplementary Material.

### Rupture potential index (RPI) assessment

RPI was evaluated throughout the surface of the AAA wall using clinical CTA images and finite element analysis (FEA) as previously described 15. Since CTA images provide visualization of AAA sac deformations under intraluminal pressure, the AAA can be treated as statically determinant, and a linear FEA can be employed to determine the stress fields across the surface of the AAA 21. AAA wall strength was calculated using 22:



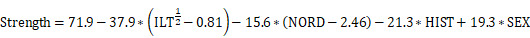



where NORD was the aneurysm diameter normalized to the infrarenal neck diameter, HIST was the family history of AAA (1 for family history, 0 otherwise), and SEX is +1/2 for male, -1/2 for female. The RPI throughout the aneurysm wall was then calculated as the stress divided by the strength. A high RPI value, e.g., around 1, indicated a higher rupture risk. The lowest RPI values in symptomatic or ruptured AAAs were reported to be as low as 0.3 23. For co-registration between RPI map and CCR2 PET/CT images, the RPI model was first mapped back onto the original CTA, with nodes in the RPI model mapped to their nearest voxel in the CTA. Voxel intensity was set relative to the RPI, and high enough to be distinct from the background CTA (**Figure S3, Supplementary Material**). These modified RPI images were then co-registered with CCR2 PET/CT images using MIM software.

### Histology, immunostaining, and zymography

Aortic wall specimens from surgical patients with AAA and controls were collected and processed for histopathological assessment, immunostaining, and zymography (**Supplementary Material**).

## Results

### Patient screening, enrollment, and demographics

**Figure 1:**
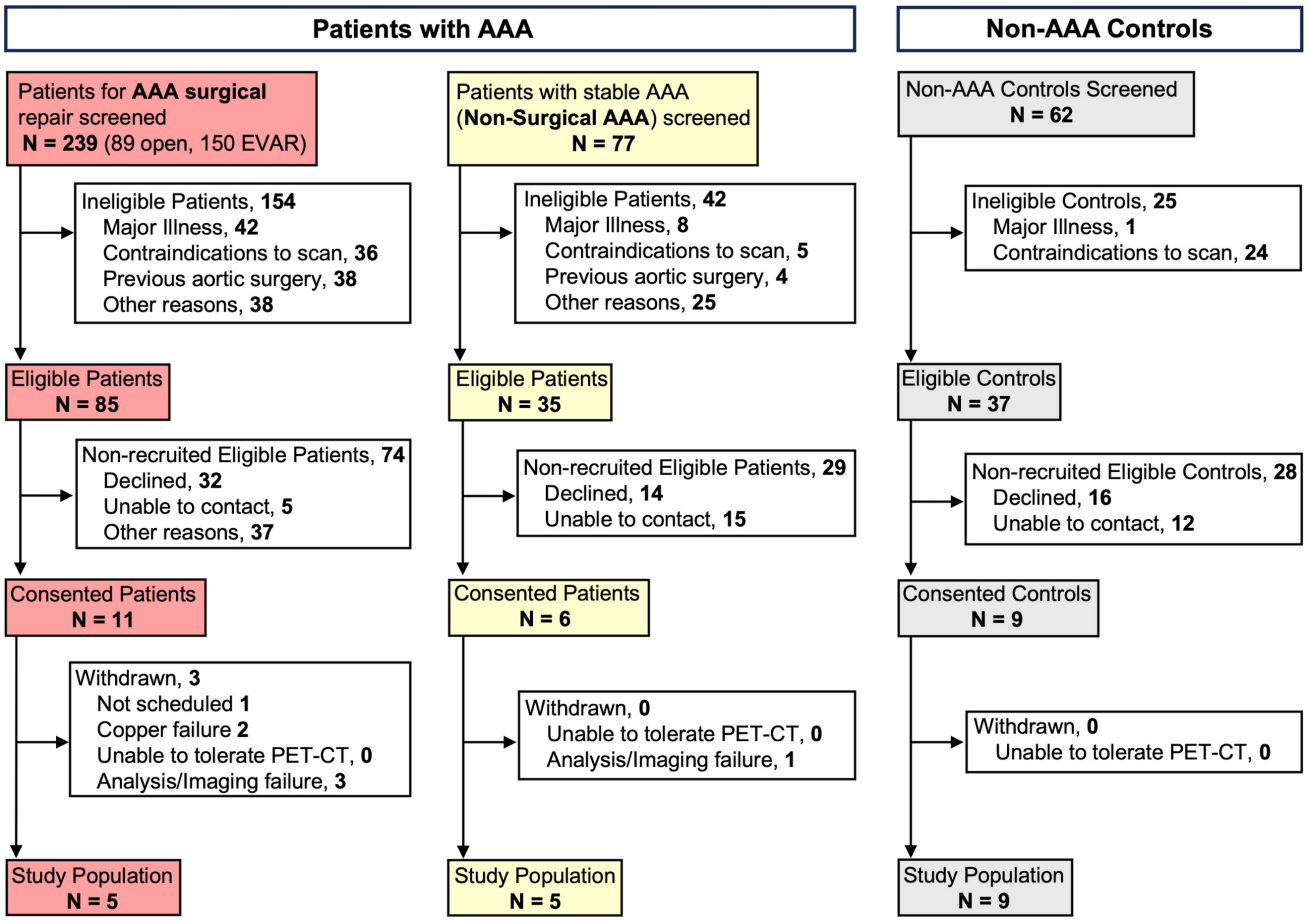
** Characteristics of individuals screened and recruited for the study.** AAA = Abdominal Aortic Aneurysm; CT = computed tomography; PET = Positron Emission Tomography; EVAR= Endovascular Aneurysm Repair.

All patients were screened based on group-specific inclusion criteria and ultimately selected to participate in the study analysis, as detailed in Supplementary Material and Figure 1. The majority of patients with AAAs were elderly, with a mean age of 70.7 ± 7.3 years, and were primarily male (70%). The average baseline diameter of the aneurysm was 4.86 ± 0.75 cm. Among the AAA patients, only three (30%) were active smokers and four (40%) were former smokers. Similarly, the non-AAA controls were predominantly early-elderly, with a mean age of 63.44 ± 13.6 years; however, this group had a lower proportion of males at 44.4%. The non-AAA controls comprised individuals who were not currently smoking, although 44.4% were former smokers (**Table 1**). All AAA and 77.8% of controls were white. Kidney function, as determined by GFR, was not significantly different between the two groups. There were similar rates of comorbidities related to AAA between groups, except for hyperlipidemia, which was significantly more prevalent in all patients with AAAs (100%) compared to only half of the control subjects (P = 0.01). None of the patients with AAAs had diabetes mellitus nor were they taking metformin. Finally, the distribution of relevant medications was similar between groups (**Table 1**).

### PET/CT imaging reveals enhanced CCR2 signal intensity in patients with AAAs

**Figure 2:**
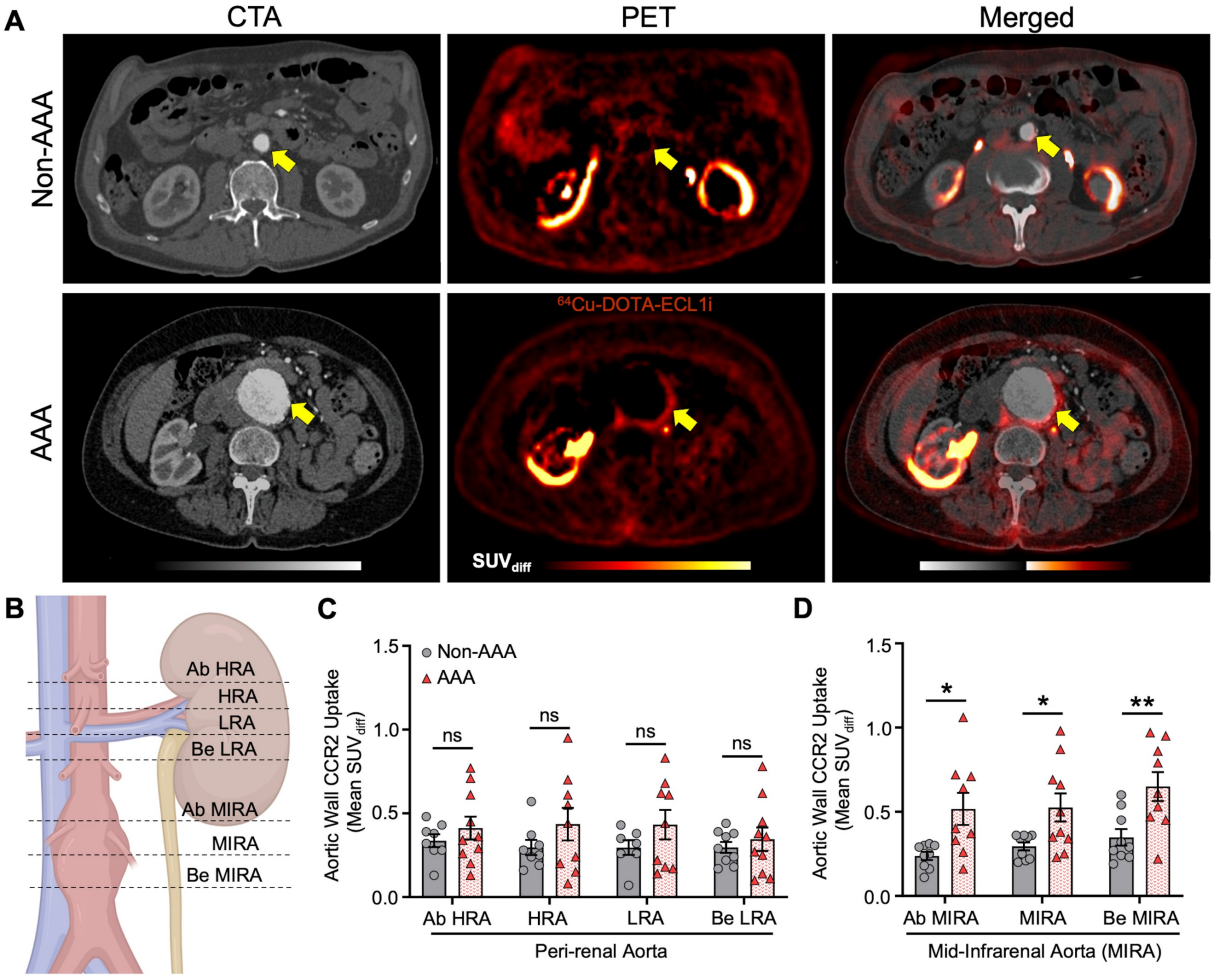
** CCR2 radiotracer [^64^Cu]-DOTA-ECL1i PET/CT signal in AAA patients and non-AAA controls**. (**A**) Representative computed tomography angiography (CTA), positron emission tomography (PET) and merged (co-registration of [^64^Cu]-DOTA-ECL1i and the anatomical structures) in AAA and non-AAA patients. (**B**) Illustration demonstrating the different anatomical slice locations evaluated, figure was made using BioRender.com. (**C**) Wall CCR2 uptake along the peri-renal and (**D**) MIRA regions in AAA and non-AAA groups. Yellow arrow = pointing to the abdominal aorta. Due to anatomical variations, CCR2 detection was not possible at specific locations in 2 non-AAA and 1 AAA patient. Ab = Above; HRA = Highest Renal Artery; Be = Bellow; LRA = Lowest Renal Artery; MIRA = Mid InfraRenal Aorta.

Compared with the non-AAA control group, the PET/CT CCR2 signal was observed within the aortic wall of patients with AAA (AAA group; **Figure 2** and **Figure S4, Supplementary Material**). PET/CT analysis using mean standardized uptake value differential (SUV_diff_) demonstrated a significant increase within the AAA group, specifically in areas of established disease such as the mid-infrarenal (MIRA) region (Ab MIRA 0.52 ± 0.3 vs. 0.24 ± 0.07 respectively, P = 0.01; MIRA 0.52 ± 0.3 vs. 0.29 ± 0.07 respectively, P = 0.02 and Be MIRA 0.65 ± 0.2 vs. 0.35 ± 0.1 respectively, P = 0.007; **Figure 2A & D**). In contrast, the peri-renal region, which did not exhibit established AAA degeneration, demonstrated an increased CCR2 signal in the AAA group, but this increase was not statistically significant (P = ns; **Figure 2A & C**).

### Surgical AAA patients exhibit a unique increase in PET/CT CCR2 signal intensity

**Figure 3:**
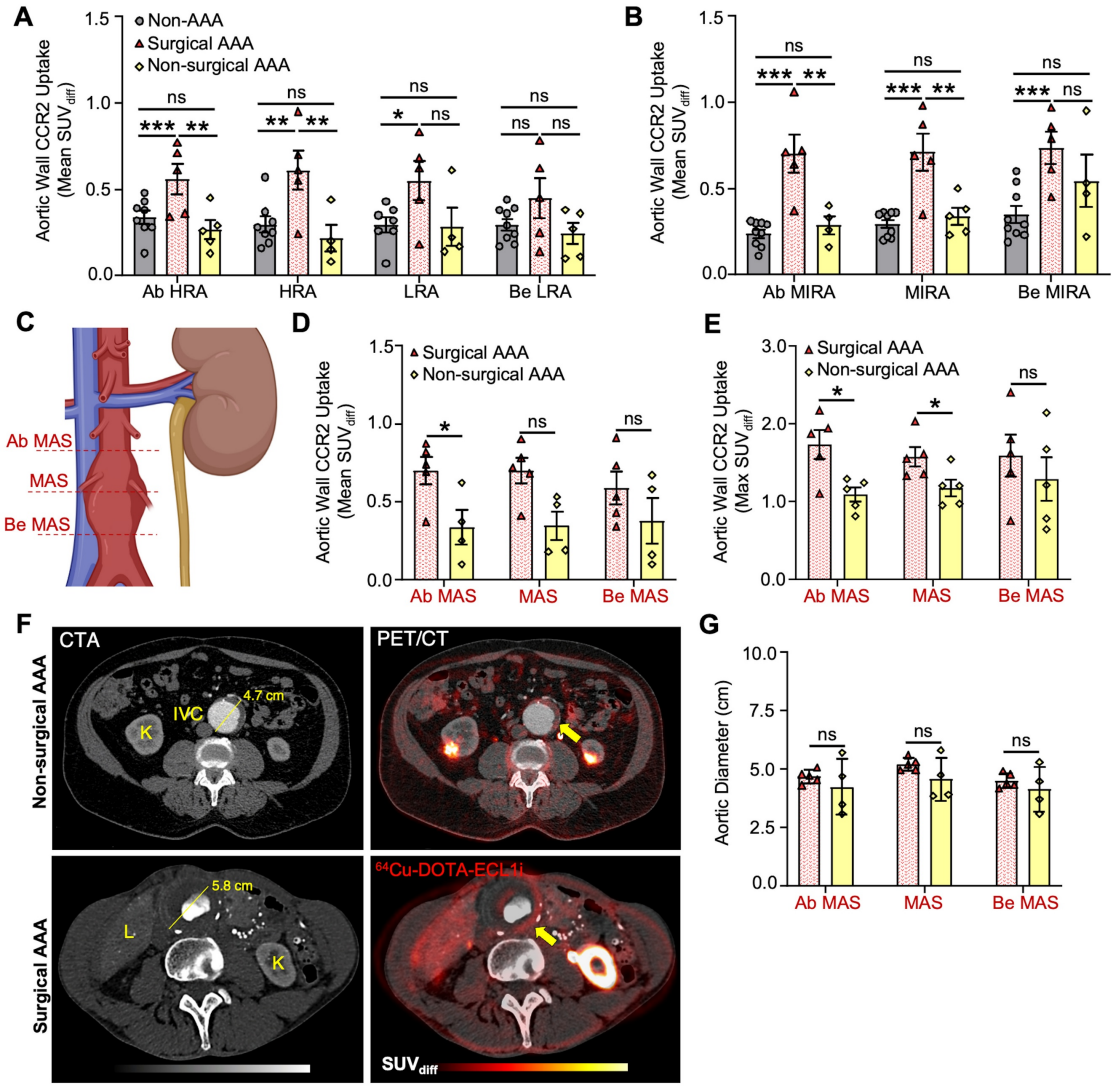
**CCR2 radiotracer [^64^Cu]-DOTA-ECL1i PET/CT signal in surgical, non-surgical AAA patients and non-AAA controls**. (**A**) Wall CCR2 uptake along the peri-renal and (**B**) MIRA region, among studied groups. (**C**) Illustration demonstrating the maximum aneurysm sac (MAS) anatomical slice locations evaluated, figure was made using BioRender.com. (**D&E**) CCR2 in terms of mean SUV_diff_ and max SUV_max_ at the MAS location between surgical and non-surgical AAAs, respectively. (**F**) Surgical and non-surgical AAA representative CTA and PET with co-registration of [^64^Cu]-DOTA-ECL1i and anatomical structures. (**G**) Aortic diameter between AAA groups. Due to anatomical variations, CCR2 detection was not possible at specific locations in 2 non-AAA and 1 non-surgical AAA patient. Yellow arrow = pointing to the Abdominal Aorta. K = Kidney; IVC = Inferior Vena Cava; L = Liver; Ab = Above; HRA = Highest Renal Artery; Be = Bellow; LRA = Lowest Renal Artery; MIRA = Mid infrarenal aorta.

Surgical AAA patients showed a larger aneurysm diameter (5.4 ± 0.23 cm) than non-surgical AAA patients (4.3 ± 0.71 cm). However, the remaining variables did not show any significant differences (**Table S1, Supplementary Material**). PET/CT analysis revealed an overall increased CCR2 signal in the aortic wall of the surgical AAA group compared to both the non-surgical AAAs and non-AAA control groups. Moreover, this increase in the signal was consistently observed across various anatomical locations and SUV interpretations (**Figure 3A & B** and **Figure S5, Supplementary Material**). Specifically, patients with surgical AAAs exhibited a modest increase in the mean SUV_diff_ CCR2 signal in the perirenal region (**Figure 3A**). A prominent more CCR2 signal was observed at the MIRA in surgical AAAs when compared to both the non-surgical AAAs (Ab MIRA 0.7 ± 0.2 vs. 0.28 ± 0.1 respectively, P < 0.001; MIRA 0.71 ± 0.2 vs. 0.34 ± 0.1 respectively, P < 0.001 and Be MIRA 0.73 ± 0.2 vs. 0.54 ± 0.3 respectively, P = ns; **Figure 3B**) and non-AAA controls (Ab MIRA 0.24 ± 0.07, P < 0.0001; MIRA 0.29 ± 0.07, P < 0.001 and Be MIRA 0.34 ± 0.1, P < 0.001; **Figure 3B)**. Interestingly, non-surgical AAAs exhibited a relatively higher CCR2 signal at the MIRA, although this was not statistically significant when compared with the non-AAA controls (**Figure 3B**). Although the AAA surgical group showed consistently elevated CCR2 tracer uptake, mild variations were observed in the signal between patients (**Figure S8A & G, Supplementary Material**). As hypothesized, CCR2 mean and max SUV_diff_ were higher in the maximum aneurysm sac (MAS) region (**Figure 3C**) in surgical AAA patients than in nonsurgical AAA patients (**Figure 3D-F**). Additionally, there was no association between relative uptake variability and aneurysm size, which remained consistent across the study groups (**Figure 3F & G**). These findings indicate that the observed differences in CCR2 PET/CT signals are independent of the aneurysm size.

### AAA histopathology correlates with CCR2^+^ macrophage infiltration

As previously described, tissue samples from surgical AAA patients in areas that demonstrated significant preoperative PET/CT CCR2 signal intensity were collected during open aneurysm repair for histopathological assessment (**Figure S6A, Supplementary Material**). Compared to non-AAA controls, immunofluorescence staining revealed significant CCR2^+^ cells (35% ± 3 vs. 8% ± 1, P < 0.001; **Figure S6C & E, Supplementary Material**) as well as CD68^+^ macrophages (31% ± 5 vs. 3% ± 1, P < 0.0001; **Figure S6C & F**) in surgical AAA specimens (**Figure S6B-F**). Interestingly, AAA surgical patients also demonstrated robust double-positive CCR2^+^ and CD68^+^ cell counts (21% ± 4 vs. 1.3% ± 0.1; P = 0.001; **Figure S6C, D & G**). CCR2^+^ macrophage tissue infiltration was associated with overall aneurysm deterioration, such as severe elastin degradation and increased MMP9 24 and MMP2 25 activity when compared to controls (**Figure S7, Supplementary Material**). Furthermore, an intragroup analysis of AAA surgical specimens collected from various regions of the aneurysm wall revealed a significant negative correlation (r = -0.85; P = 0.006) between DAB staining intensity of CCR2 and VVG-stained elastin fiber content (**Figure S7E & F**). CCR2 staining also demonstrated a positive, but non-significant, correlation with MMP9 activity in the aortic wall (r = 0.33; **Figure S7G**). These finding suggest that CCR2 may contribute to AAA wall instability.

### CCR2 radiotracer signal in areas of aortic wall stress

**Figure 4:**
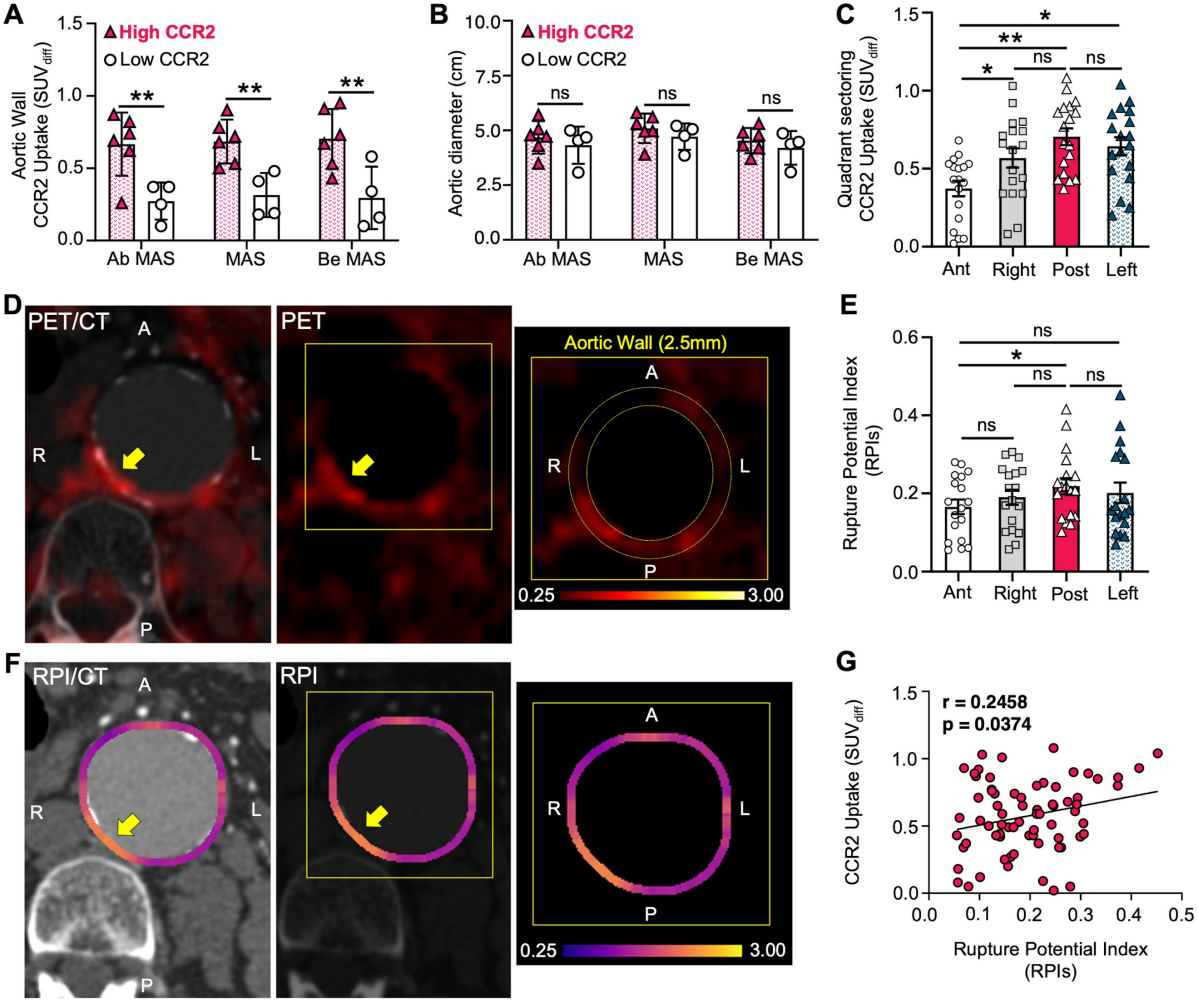
** High CCR2 AAA group correlates to RPI.** (**A**) Differences in CCR2 uptake in terms of mean SUV_diff_ within the MAS area between High and Low AAA groups, (**B**) irrespective of aneurysm diameter. (**C**) CCR2 signal uptake in aortic wall relative to quadrants. (**D**) Representative example of CTA and PET imaging in High AAA group patient with CCR2 tracer signal uptake in the posterior quadrant. The yellow arrow pointing to the AAA and the zoomed-in image demonstrates the quadrant sectoring. (**E & F**) Peak RPI was found in the posterior aortic wall. (**G**) CCR2 signal and RPI in all quadrants demonstrated a significant correlation.

There was a positive, non-significant correlation between PET/CT CCR2 radiotracer signal and RPI in both the surgical and non-surgical AAA groups (r = 0.18 and r = 0.37, respectively), with the highest RPI of the AAA wall, as detailed in **Supplementary Material**, **Figure S8**. To further evaluate the ability of the CCR2 radiotracer to predict AAA wall stress, we used the AAA wall cumulative uptake value (0.53±0.3) within the MAS area as a threshold for high or low signal classification in order to subdivide all AAA patients into two groups (**Supplementary Results 6**; six high CCR2 patients and four low CCR2 patients). CCR2 radiotracer signal quantification, represented by mean SUV_diff_, revealed higher radiotracer signals in Ab MAS, MAS, and Be MAS segments of patients with high CCR2 levels (P = 0.002, P = 0.002, and P = 0.004, respectively; **Figure 4A**). Notably, no differences were observed in AAA diameter (**Figure 4B**) or demographics between the two groups (**Table S2**).

The high CCR2 group demonstrated elevated CCR2 radiotracer signal intensity in the posterior quadrant of the AAA wall, followed by the left, right, and anterior quadrants (P = 0.006; **Figure 4C & D**). Similarly, the mean RPI values peaked in the posterior quadrant, followed by the left, right, and anterior quadrants, respectively. Notably, the only significant difference was observed between the posterior and anterior quadrants (P = 0.037; **Figure 4E & F**). Furthermore, we found a significant direct correlation between CCR2 signals and RPI values, suggesting that the radiotracer has the potential to identify regional differences in aortic wall stress and vulnerability (P = 0.037; **Figure 4G**).

### Prospective Follow-Up Analysis of AAA Patients

To evaluate the predictive ability of the PET/CT CCR2 radiotracer, we conducted a prospective assessment tracking all-cause mortality, AAA-related events (ruptures, surgical repairs, and post-EVAR endoleaks), and mean AAA growth (**Table S3**). At 6 months and 1 year, no all-cause mortality or major morbidity events (such as myocardial infarction or stroke) were observed. In the AAA surgical group, only one patient experienced an endoleak post-EVAR. In contrast, the non-surgical AAA group experienced four AAA-related events, including two EVARs, both resulting in endoleaks; one of these was in the high CCR2 group, and the other was in the low CCR2 cohort. Furthermore, the mean AAA diameter growth at 6 months and 1 year was 0.7 mm and 5 mm, respectively. Notably, the high CCR2 subgroup exhibited greater mean growth (1.9 mm and 10 mm) compared to the low CCR2 group (0.3 mm and 1 mm) over the same periods. Although this is an early trend, this finding suggests more significant aneurysm growth in the high CCR2 subgroup. The small sample size precludes statistical significance at this time.

## Discussion

Here we present a prospective pilot case-control clinical study demonstrating the potential of molecular imaging targeting of CCR2 in AAA progression and its correlation with wall instability. Using the CCR2 PET/CT radiotracer, we observed that CCR2 signal intensity increased in the aortic wall of AAA patients, particularly in those with advanced aneurysmal disease. This aligns with previous findings that have evaluated the role of CCR2 in vascular inflammation and the risk of AAA rupture 18,19. Notably, surgical AAA patients demonstrated higher CCR2 signals irrespective of aneurysm size, linking elevated CCR2 activity to disease severity and aggressive inflammation. Furthermore, histopathological analysis of surgical AAA samples collected during open repair confirmed the substantial infiltration of CCR2+ macrophages. This finding aligns well with prior studies showing that CCR2 content in AAA tissue correlates with the severity of elastin degradation and metalloproteinase activity, a classic hallmark of AAA disease severity 26. Interestingly, we observed regional differences in CCR2 signal intensity within the MAS, demonstrating variability in inflammation in aneurysmal tissue. The highest CCR2 PET/CT signal was unequivocally detected in the MAS region, particularly in the posterior quadrant of the aortic wall. Additionally, a subset of AAA patients exhibited both high CCR2 signals and high RPI values, demonstrating a significant correlation. This suggests that CCR2 PET/CT may play an important role in identifying mechanical wall stress and instability independent of aneurysm size and diameter. Lastly, the prospective follow-up analysis showed a trend of increased mean AAA growth in the high CCR2 signal group, further suggesting the potential predictive ability of the tracer.

Our team recently reported that CCR2 may serve as a key theranostic biomarker for AAA progression and rupture 18. In preclinical models, pharmacological CCR2 inhibition reduced AAA expansion and fatal rupture rates, leading to our first-in-human clinical evaluation of CCR2-targeted imaging in AAA patients. Our initial study of four healthy and four AAA patients demonstrated increased CCR2 PET/CT 18. In this expanded analysis of 19 subjects, we implemented an advanced PET/CT methodology to enhance location specific signal quantification accuracy, minimized imaging artifacts, and performed unbiased anatomical segmentation while also correcting for intraluminal blood volume signal. We further incorporated aortic wall layer-specific and quadrant-based analyses while correlating radiotracer uptake with histological findings and RPI.

There is a critical need for reliable imaging protocols using cellular and molecular markers to assess patient-specific aneurysm characteristics, given the limited ability of the AAA diameter to predict the risk of rupture 27. PET/CT imaging with novel radiotracers such as CCR2-targeting [^64^Cu]-DOTA-ECL1i offers a potentially novel and non-invasive method for visualizing and quantifying inflammation within the AAA wall tissue. This would provide valuable insights beyond traditional imaging modalities such as CTA and magnetic resonance imaging (MRI). The role of CCR2 in AAA has been highlighted in several studies 17-19,28, demonstrating its potential as a theranostic marker of AAA disease progression. Our study findings suggest that CCR2 PET/CT molecular imaging may be a valuable tool for assessing the extent of AAA as well as regions with elevated rupture risk. Identifying high-risk AAA patients has the potential to enhance and personalize disease management for large segments of patients that are typically managed expectantly until their aneurysms increase in size, become more complex to repair, or progress to rupture.

The majority of PET/CT studies that have evaluated AAA disease have primarily focused on the use of ^18^F-Fluorodeoxyglucose (^18^F-FDG), a radiotracer that highlights metabolic activity by tracking glucose uptake in various cells and tissues 28. Although studies using ^18^F-FDG have consistently demonstrated encouraging correlations with AAA histopathology, clinical events, and aneurysm expansion, its clinical utility remains controversial 27,28. Some studies have reported increased ^18^F-FDG uptake in patients with AAAs that progress to adverse clinical events, whereas others have found decreased uptake in these patients 28. While some pre-clinical 29,30 and clinical studies 31 have suggested that ^18^F-FDG signal correlates with tissue inflammation, these findings are inconsistent when using simultaneous MRI assessments and other molecular imaging tools when assessing chronic inflammation 32,33,34. One potential explanation for this variation is that ^18^F-FDG lacks the sensitivity to accurately evaluate the extent of inflammatory cell infiltrates in tissue, thereby offering limited objective imaging indicators for the prognosis of AAAs.

In a landmark investigation, Xu et al. were the first to demonstrate that high aortic wall stress, using RPI values (calculated via the finite element methods described by Joldes et al. 31), correlated with areas of positive ^18^F-FDG uptake 35. Furthermore, these results were consistent with the findings of Nchimi et al., who observed an even stronger correlation in thoracic aortic aneurysms than in AAAs 36. These findings suggest a potential link between accelerated metabolism within the AAA wall (^18^F-FDG uptake) and high mechanical wall stress, which could aid in diagnosis and further patient stratification. Similarly, we observed a significant correlation between CCR2 radiotracer signal intensity and RPI (**Figure 4**). These observations appear to be independent of aneurysm diameter, at least within the range of aneurysm sizes included in our study, and are strongly supported by prior research using the (^18^F-FDG) tracer 11,37. Moreover, our study included patients with an average aneurysm diameter of 4.86 cm, which is below the typical threshold for surgical intervention and significantly lower than the 7 cm diameter previously associated with a higher probability of rupture 13. This smaller average aneurysm size may have limited our ability to observe a correlation between aneurysm disease severity and radiotracer signal. Nevertheless, this is the first human trial to show that cellular and molecular markers, such as CCR2 are correlated with AAA wall instability. In this instance, the radiotracer demonstrates a distinct advantage over ^18^F-FDG in its ability to differentiate disease-specific activity and potentially identify selected groups of patients for targeted anti-inflammatory medical therapy and surgical decision-making.

Recent findings from a prospective multi-center study, the SoFIA study, which utilized a novel fluorine-18-sodium fluoride [^18^F-NaF] PET/CT to assess AAA progression and rupture, demonstrated efficacy of this tool in identifying disease activity in humans 38. This study correlated tracer uptake with areas of microcalcification, suggesting the tracer's potential role as a susceptibility marker for aneurysm expansion and rupture. Furthermore, the trial showed that tracer uptake is independent of the aneurysmal diameter and serves as a valuable supplement to traditional clinical parameters 38. In contrast, our study suggests that CCR2 radiotracer signal may be a better predictor for assessing or quantifying the risk of AAA rupture. This observation provides notable utility, especially since the CCR2 radiotracer signal in the aortic wall appears to be significantly correlated with mechanical stress, as demonstrated in **Figure 4**. Moreover, the SoFIA study did not provide information on the exact regions traced or the specific methods used to eliminate radiotracer spillover contamination. According to their methodology, “care was taken to exclude tracer uptake that originated from nearby bone structures or from the urinary tract 38.” On the other hand, our study used a rigorous protocol, including dynamic and static PET/CT imaging, along with precise anatomical localization and segmentation to help ensure accurate and reproducible results (**Supplementary Material**). Moreover, our study developed a novel SUV_diff_ technique for the analysis of tracer uptake and heterogeneity within the aortic wall that was able to reduce the blood pool signal and spill-over contamination (**Figure S1 & S2**). A caveat of our technique of spill-over correction is that it does not include correction for partial volume that would address the partial recovery of the activity in the aortic wall. Further validation with phantom studies and simulations are currently underway to address this limitation.

Our study is among the first to use PET/CT imaging to assess CCR2 signal intensity in both AAA and non-AAA patients. We included groups of surgical and non-surgical AAA patients, as well as non-AAA controls, providing a comprehensive overview of CCR2 activity across different patient populations. The prospective design of our study ensured systematic data collection and analysis, thus enhancing the robustness of our findings. By integrating PET/CT imaging with histopathological analysis, we have gained a deeper understanding of the mechanistic role of CCR2 in AAA, thereby increasing the validity of our imaging results. Nevertheless, we acknowledge several limitations to our study. First, being a single-center study, the generalizability of our findings to other populations and clinical practice settings is limited. Importantly, the relatively small number of patients in each group may have reduced the statistical power and the ability to detect smaller differences. As a pilot and first-in-human study, we focused on evaluating the feasibility of *in vivo* PET/CT imaging of CCR2 in AAAs without stratifying patients into medical observation versus surgical treatment groups. Consequently, although prospective follow-up demonstrated an early trend of increased AAA growth in the high CCR2 signal group (**Table S3**), the lack of sufficient longitudinal aneurysm rupture outcome data limits the statistical significance of our findings. Additionally, although we focused primarily on CCR2 signaling, this approach potentially overlooked other relevant inflammatory pathways and factors that likely also contribute to AAA progression and rupture. Despite the advanced nature of PET/CT imaging, inherent limitations related to resolution and specificity might have affected the accuracy of CCR2 signal measurements, which we have tried to mitigate routinely with our various analytical approaches. Additionally, patient anatomical variations sometimes prevented us from gathering SUV uptake data from specific locations within the restricted PET/CT segment. This limitation was due to both anatomical differences and our goal to minimize radiation exposure. Future studies will aim to develop a larger multicenter study to further validate our findings and broader clinical applicability.

## Supplementary Material

Supplementary Methods, Results, Figures S1-S8 and Tables S1- S3. Supplementary figures and tables.

Supplementary video 1.

## Figures and Tables

**Table 1:** Demographics for individuals with no AAAs and patients with AAAs.

		Non-AAA Control (N = 9)	AAA (N = 10)	P value
**Age** mean (SD)		63.44 (13.6)	70.70 (7.3)	0.16
**Sex** (Male)		4 (44.4%)	7 (70%)	0.26
**Race**	White	7 (77.8%)	10 (100%)	0.28
	African American	1 (11.1%)	0	N/A
	Asian	1 (11.1%)	0	
**Smoking Status**	Former	4 (44.4%)	4 (40%)	0.17
	Active	0	3 (30%)	N/A
**GFR** mean (SD) mL/min		73 (19)	73 (23)	0.98
**Aneurysm Diameter** mean (SD)		N/A	4.86 (0.75)	N/A
**Concurrent Iliac Aneurysm**		N/A	4 (21.1%)	N/A
**Comorbidities**	Hypertension	6 (75%)	9 (90%)	0.39
	Diabetes Mellitus	1 (12.5%)	0	0.25
	Hyperlipidemia	4 (50%)	10 (100%)	**0.01**
	Ischemic Heart Disease	2 (25%)	4 (40%)	0.50
	Peripheral Artery Disease	0	1 (10%)	0.35
	Cerebrovascular Disease	0	5 (50%)	**0.01**
**Medications**	Metformin	0	0	N/A
	Statin	4 (50%)	9 (90%)	0.06
	Beta blocker	2 (25%)	1 (10%)	0.39
	CCB	4 (50%)	5 (50%)	1.00
	ACEI/ARB	4 (50%)	6 (60%)	0.67
	Antiplatelet	5 (62.5%)	6 (60%)	0.91
	Anticoagulant	0	2 (20%)	0.18

AAA =Abdominal Aortic Aneurysm; GFR = Glomerular Filtration Rate; CCB = Calcium Channel Blocker; ACEI = angiotensin-converting enzyme inhibitor; ARB = angiotensin receptor blocker; N/A = non-applicable; SD = Standard Deviation.

## Abbreviations

CCR2: C-C chemokine receptor type 2
PET/CT: Positron Emission Tomography/Computed Tomography
AAA: Abdominal Aortic Aneurysms
RPI: Rupture Potential Index
DOTA: Tetraazacyclododecane tetraacetic acid
ECL1i: extracellular loop 1 inverso
Cu: copper
MMP: matrix metalloproteinases
CTA: Computed Tomography Angiogram
FEA: finite element analysis
ILT: Intraluminal Thrombus
GFR: glomerular filtration rate
CCB: calcium channel blocker
ACEI: angiotensin-converting enzyme inhibitor
ARB: Angiotensin receptor blocker
N/A: non-applicable
SD: Standard Deviation
EVAR: Endovascular aneurysm repair
SUV: Standardized uptake value
MAS: Maximum Aneurysm Sac
HRA: Highest Renal Artery
LRA: Lowest Renal Artery
MIRA: mid-infrarenal aorta
MRI: magnetic resonance imaging
FDG: Fluorodeoxyglucose
