# Supplementary material for: Pilot first-in-human CCR2 PET/CT to detect abdominal aortic aneurysm wall instability
Source: Theranostics. 2025 Apr 13;15(12):5518–28. doi: 10.7150/thno.108656 (PMC12068289; doi:10.7150/thno.108656)
Supplement: Supplementary file 1 — Supplementary Methods, Results, Figures S1-S8 and Tables S1- S3. Supplementary figures and tables. [file thnov15p5518s1.pdf]

Pilot First-In-Human CCR2 PET/CT to Detect Abdominal Aortic Aneurysm Wall Stress

SUPPLEMENTARY MATERIAL

Authors

Santiago Elizondo-Benedetto[1*], Deborah Sultan[2*], Ryan Wahidi[1], Mahdjoub Hamdi[2], Mohamed S. Zaghloul[1], Shahab Hafezi[1], Batool Arif[1], Laura K. McDonald[1], Kitty Harrison[2], Dakkota Thies[2], Gyu Seong Heo[2], Hannah Luehmann[2], Lisa Detering[2], J. Westley Ohman[1], Zachary J. Wanken[1], Luis A. Sanchez[1], Joseph E. Ippolito[2,3], Jie Zheng[2], Robert J. Gropler[2], Richard Laforest[2], Yongjian Liu[2], Mohamed A. Zayed[1-2,4-7]

Affiliations

[1] Section of Vascular Surgery, Department of Surgery, Washington University School of Medicine, St. Louis, MO, USA.
[2] Department of Radiology, Washington University School of Medicine, St. Louis, MO, USA.
[3] Department of Biochemistry and Molecular Biophysics, Washington University School of Medicine, St. Louis, MO, USA.
[4] Division of Molecular Cell Biology, Washington University School of Medicine, St. Louis, MO, USA.
[5] Division of Surgical Sciences, Department of Surgery, Washington University School of Medicine, St. Louis, MO, USA.
[6] Department of Biomedical Engineering, McKelvey School of Engineering, Washington University School of Medicine, St. Louis, MO, USA.
[7] Veterans Affairs St. Louis Health Care System, St. Louis, MO, USA.

Address for Correspondence:
Mohamed A. Zayed, MD, PhD, MBA
660 S. Euclid Avenue
Campus Box 8109 - Surgery
St. Louis, MO, USA
E-mail: zayedm@wustl.edu

* These authors contributed equally to this work

<u>Supplementary Methods</u>

1. Study population, eligibility criteria and protocol specifics

2. PET/CT imaging and analysis protocol

3. Histology, immunostaining and zymography

<u>Supplementary Results</u>

4. Patient screening, enrollment and demographics

5. Association between PET/CT radiotracer signal and RPI in AAA tissue

6. High vs Low CCR2 signal threshold analysis

7. Prospective follow-up of AAA patients: mortality events, AAA-related complications and aneurysm expansion

1. Study population, eligibility criteria, and protocol specifics

Patients were identified from the Washington University/Barnes-Jewish Hospital vascular surgery outpatient clinics and were screened by research coordinator personnel. Exclusion criteria included inability to provide informed consent, chronic renal insufficiency with a glomerular filtration rate (GFR) < 30, documented allergies to contrast media, hemodynamic instability, inability to remain in a supine position for 60 minutes required for PET/CT and CTA, a positive pregnancy test or current lactation, and cancer conditions requiring oncologic management. Study eligible patients were contacted by members of the study team to discuss the research protocol in detail prior to obtaining written consent for study participation. The study was conducted in compliance with the protocol approved by Washington University Institutional Review Board, ID: 202008190, the Human Research Protection Office, and in accordance with state and federal government regulations standards of Good Clinical Practice and applicable institutional research policies and procedures. Eight patients (4 healthy volunteers, and 4 AAA patients) were previously reported [18].

2. PET/CT imaging and analysis protocol

[^{64}Cu] Cu-DOTA-ECL1i PET-CT Imaging. All PET/CT studies were performed on Siemens Biograph Vision-600 in the Center for Clinical Imaging Research at Barnes-Jewish Hospital. An initial low dose CT was performed for attenuation correction, followed by an injection of 9.3 (±0.6) mCi of [^{64}Cu] Cu-DOTA-ECL1i, followed by a 60-minute dynamic PET/CT. A CTA angiogram was acquired if the patient's previous CTA was older than two weeks. The PET/CT scan was then elastically registered to the CTA for accurate anatomical localization. The summed PET images from 30-60 minutes were reconstructed and evaluated on a 5-point scale for quality

purposes. Semi-quantitative analyses, such as standardized uptake value (SUV), mean and max were also generated.

Static PET/CT scan image analysis. We used Standardized Environmental for Radiomics Analysis (SERA) software that proposes up to 487 radiomics measures and is capable of processing images from various clinical imaging modalities such as PET and CT in compliance with IBSI and ensures data reproducibility. PET images were reconstructed with maximum a posteriori algorithm and quantified as mean and max static SUV (SUV_s) from regions of interest (ROI) using MIM software (MIM Software, Inc.; Beachwood, Ohio). Fractional lesion volume of tracer uptake as determined by the number of voxels above threshold technique from a three-dimensional volume-of-interest (VOI) was ascribed in aortic ROIs as correlated on corresponding CTA. An optimal threshold was determined from a histogram-based analysis to discriminate blood activity. This information provided a global measure of the lesion burden. Furthermore, radiomics measures of heterogeneity were evaluated to express the level of dispersion or clustering of radiotracer signal within the VOI. Using a standard library of features taken from the Image Biomarker Standardization Initiative (IBSI; https://arxiv.org), we extracted radiomics features related to morphology, local intensity, and intensity base volume-histogram features, as well as gray-level co-occurrence features as examples.

CTA assessment. Utilizing a Vitrea 3D workstation (Canon Medical Systems Corporation: Otawara, Japan), CTA studies were analyzed using centerline reconstruction to obtain maximum AAA diameters, and AAA sac expansion rates were derived from comparisons to prior CTAs. In addition, thrombus-to-AAA volume ratios were evaluated. Aortic neck diameter, length, and α-angles were measured, and iliac artery tortuosity indices, common iliac artery diameters, and iliac bifurcation angles were also measured.

PET imaging blood subtraction analysis. To determine the radiotracer signal in aortic wall tissue, radiotracer signal in early frames in the blood pool were subtracted from the integral static PET image to isolate the radiotracer signal within the wall of the aorta. This was done using the time activity curve (TAC) in the inferior vena cava (IVC) to visually select early and late frames (**FigureS1A**). These frames were time-averaged, resulting in 2 PET images (SUV_{early} and SUV_{late}) and a scaling factor. These were used to provide a differential SUV (SUV_{diff}), which is a measure of the residual activity in the aortic wall tissue after correction for effective radiotracer signal spill-over (accounting for PET/CT spatial resolution) and subtraction of the first pass blood perfusion in aortic tissue. The final differential SUV (SUV_{diff}) image was obtained using the following formula:

$$SUV_{diff} = SUV_{late} - F\ SUV_{early}$$

where F is calculated as the ratio of SUV values in the blood pool (from either descending aorta or vena cava) at late over early time points. The temporal definitions of the early and late time points are indicated in **Figure S1**. Residual negative SUV_{diff} values in blood pool areas were set to zero in the final SUV_{diff} images used for analysis of the radiotracer signal in AAA tissue. Both SUV_{diff} and SUV_s were assessed and compared throughout the analysis.

Aortic anatomical segmentation. In 3 studied anatomical regions (para-renal, mid-infrarenal, and maximum aneurysm sac) a total of 10 anatomical slice locations were selected on the CTA to measure the SUV signal in all study participants. Based on anatomical landmarks, anatomical slice locations were defined as: highest renal artery (HRA) (either the right or left renal artery), as well another at 10 mm or slice cut above the recently mentioned (Ab HRA). Similarly, the

lowest renal artery (LRA) and a 10 mm or slice cuts below (Be LRA) were defined. Mid-infrarenal aorta (MIRA) slice location was defined as the equidistant segment between the LRA and the aortic bifurcation, 10 mm, or slice cuts above (Ab MIRA) and below (Be MIRA) were also considered. Lastly, the maximum aneurysm sac (MAS) was defined as the largest aneurysmatic diameter for each patient and was therefore considered as the most diseased region; 10 mm or slice cuts above (Ab MAS) and below (Be MAS) were also considered (**Figure S2A-C**). Using MIM software analysis tool (2D brush), aortic structures were outlined and isolated into segments of interest (SOIs; **Figure S2D** and **Video S1**). The aortic wall was defined and traced at 2.5mm thickness for all study participants. The aortic lumen was also defined, as well as the intraluminal thrombus (ILT) if encountered. A 5mm thickness outer ring from the external border of the aortic wall was also selected and identified as peri-aortic adventitia. Using MIM software analysis tool (simple sectors), was also used to perform a quadrant sectoring for 2 SOIs (aortic wall and peri-aortic tissue) and defined as anterior, posterior, left, and right quadrants (**Figure S2E**).

Statistics. All data are presented as the mean ± SD. For the analysis of continuous variables, most group comparisons were performed using an unpaired t-test. For comparisons that included one endpoint in more than one group, an ordinary one-way ANOVA with multiple comparison was performed. For comparisons that included more than one endpoint in more than one group, we utilized a two-way ANOVA with multiple comparison. For the analysis of categorical variables, all group comparisons were performed using Chi-Square test to assess associations between these variables. For correlation analysis Pearson was employed for approximately normally distributed continuous variables while Spearman was performed when data did not meet assumptions of normality. VVG cross section staining's were analyzed using ImageJ

software by color deconvolution, adjust threshold and region of interest assessment of the AAA to analyze elastin degradation, as previously performed [17]. Immunofluorescence in AAA wall tissue-stained with CD-68 (red-channel), CCR2 (green channel), and DAPI (blue channel) was analyzed and quantified using Leica LAS X software. Data was considered statistically significant with $p \leq 0.05$. GraphPad Prism 10 (La Jolla, CA) and IBM SPSS 23 (IBM Corp., Armonk, NY) were used for all statistical analyses and graphical data representations.

3. Histology, immunostaining and zymography

Aortic wall specimen collection, processing, and storage. Aortic wall biopsies (~1 cm wide) were obtained from surgical AAA patients, and the operating surgeon provided approximate coordinates of the biopsy relative to the lowest renal artery and iliac bifurcation. Biopsies utilized for nucleic acid analyses were frozen in liquid nitrogen and stored in a dedicated - 80°C freezer. Remaining tissues were collected from the operating room in cold saline and immediately transferred on ice to an institutional vascular tissue biobank for tissue processing and analysis. The pieces of specimens were fixed in HistoChoice (VWR Life Science; Radnor, Pennsylvania), paraffin embedded, and stored at room temperature until further sectioning and staining. Aortic specimens were also frozen, embedded in optimal cutting temperature (OCT) compound, and stored at -80°C. Specimens were divided in a consistent fashion from cephalad to caudal, and the dimensions of each piece were recorded to substantiate the approximate anatomic location of the specimen for future correlation with inflammation and radiotracer signal.

Histopathology and immunostaining. For histopathological assessment, aneurysmal tissue samples were collected from 3 surgical AAA patients during open aneurysm repair, while 3 non-AAA aortic tissues were also collected from consented abdominal organ donors and used as

controls. A total of 9 samples were derived from the surgical group, from different segments of the AAA wall. To evaluate AAA tissue morphology and pathology, tissue sections were evaluated using hematoxylin and eosin (H&E) and Verhoeff-Van Gieson (VVG). All sections were imaged using Hamamatsu NanoZoomer HT 2.0 (Hamamatsu Photonics; Shizuoka, Japan), supported by the Alafi Neuroimaging Laboratory (Washington University School of Medicine). To further correlate the CCR2 role in AAA pathology, processing for antigen retrieval was performed with sodium citrate solution, pH 6.0, for 10 minutes. Tissue sections were blocked with 10% serum, and sections were incubated with primary antibody anti-CD68, 1:100 (Bio-Rad Laboratories; Hercules, California, MCA341GA) and anti-CCR2, 1:200 (Novus-Biologicals, LLC; Centennial, Colorado, NBP1-48338). For immunofluorescence, sections were incubated with donkey anti-mouse (Alexa Fluor 647), and donkey anti-rabbit (Cy3) (Jackson ImmunoResearch Laboratories). For Diaminobenzidine (DAB) immunostaining, sections were incubated with anti-rabbit secondary antibodies conjugated with HRP (Cell Signaling), DAB peroxidase substrate kit (Vector Laboratories), and counter stained with hematoxylin. All sections were imaged using Leica THUNDER Imager 3D tissue microscope system.

MMP activity zymography. For each AAA and non-AAA tissue sample, 25μg of protein was loaded on wells of 10% gelatin zymogram electrophoresis gels. Gels were then incubated in zymogram renature buffer for 30 minutes, followed by 36 hours of zymogram development buffer at 37°C. Gels were then stained with Coomassie Brilliant Blue R-25 solution from BioRad for 30 minutes, followed by destaining buffer (20% methanol, 20% acetic acid, 60% DI water) until MMP bands were visualized. Gels were scanned on BioRad Chemi doc and analyzed using ImageJ software.

4. Patient screening, enrollment and demographics

A total of 239 individuals with AAAs were screened for inclusion into the surgical AAA group. Of these, 74 were approached for participation, and 8 patients ultimately presented to the PET/CT imaging session; 2 patients could not be scheduled by the time of the analysis and 1 patient was excluded from the final analysis due to comorbidity interfering with radiotracer uptake, leaving 5 remaining patients (4 open and 1 endovascular aneurysm repair; EVAR). A total of 77 patients with stable AAAs (non-surgical AAA) were screened for inclusion: 35 were approached and 6 patients ultimately presented to the imaging session. One patient was excluded from the final analysis due to comorbidity interfering with radiotracer uptake and RPI assessment, leaving a total of 5 patients. Additionally, a total of 62 volunteers (non-AAA controls) were screened for inclusion: 28 were approached and 9 were ultimately scanned and included in the study population (**Figure 1**).

5. Association between PET/CT radiotracer signal and RPI in AAA tissue

PET/CT CCR2 radiotracer signal intensity in the surgical and non-surgical AAA study participants was compared (**Figure S8**). MIRA and MAS had the highest signal when compared to the peri-renal region (**Figure S8B & H**). Within the MAS region, the wall, ILT, and periaortic SOIs showed similar values and demonstrated significantly higher signal compared to the lumen ($P < .01$; **Figure S8C & I**). Interestingly, the posterior quadrant of the aortic wall had the highest signal, followed by the left and right, but this was statistically significant only when compared to the anterior quadrant ($P = 0.02$; **Figure S8D & J**). The RPIs followed a similar pattern, with the posterior quadrants demonstrating the highest RPI, followed by the left, right, and anterior quadrants; hence, all RPIs were significantly increased compared to the anterior quadrant (P <

0.01; **Figure S8E & K**). Additionally, we found a positive correlation between CCR2 signal intensity using mean SUV_{diff} and RPI mean values in each segment and quadrant within the MAS area in both the surgical (r = 0.18; **Figure S8F**) and non-surgical AAA groups (r = 0.37; **Figure S8L**).

6. High vs Low CCR2 signal threshold analysis

To evaluate the PET/CT SUVdiff values in AAA patients, we focused on the maximum aneurysm sac (MAS) area, including anatomical slices 1 cm above (Ab MAS), at the MAS, and 1 cm below (Be MAS). Each patient's PET/CT signal was averaged across these regions, and the resulting values were then averaged across all patients (N=10). The cumulative CCR2 uptake at MAS for all AAA patients was 0.53 ± 0.2, establishing a threshold for classifying the signal as high or low. Six patients exhibited average MAS CCR2 PET/CT signals above this threshold (with individual values of 0.57 ± 0.3, 0.65 ± 0.1, 0.72 ± 0.03, 0.73 ± 0.3, and 0.84 ± 0.06), while four patients had signals below the threshold (with values of 0.13 ± 0.05, 0.20 ± 0.04, 0.37 ± 0.03, and 0.48 ± 0.1).

7. Prospective analysis of AAA surgical patients: mortality, AAA-related outcomes and aneurysm growth

To explore the early predictive ability of CCR2 PET/CT radiotracer for the risk of AAA rupture or expansion, we decided to prospectively assess survival in terms of mortality rate, AAA-related events, and AAA growth to identify any potential trends. To date, there have been no reported cases of all-cause mortality or major morbidity events, such as myocardial infarction and strokes (**Table S3**). Specifically, prospective follow-up revealed zero mortality and morbidity events at 6

months (9/10 patients) and at 1 year (8/10 patients). AAA-related events, including ruptures, surgical repairs and post-EVAR endoleaks, are also detailed in Table S3. In the surgical AAA group (N=5), there was only one AAA-related event: an endoleak in an EVAR-repaired patient. Conversely, the non-surgical AAA group (N=5) experienced a total of four AAA-related events. This included two surgical repairs (both EVARs), each of which resulted in an endoleak. Notably, one of these patients belonged to the high CCR2 group, while the other was part of the low CCR2 cohort (**Table S3**). Furthermore, we prospectively measured mean AAA growth in non-surgical AAA patients at 6 months and 1 year after the baseline CCR2 PET/CT study. The mean diameter growth of AAA was 0.7 mm and 5 mm at 6 months and 1 year, respectively. Interestingly, the high CCR2 sub-group demonstrated a greater mean aneurysmal growth of 1.9 mm and 10 mm at 6 months and 1 year, respectively, compared to the low CCR2 group, which showed growth of 0.3 mm and 1 mm over the same periods (**Table S3**). Although there is a trend suggesting that the high CCR2 sub-group may experience more aneurysm growth, we cannot draw definitive conclusions due to the small sample size, which precludes statistical testing. Additionally, aneurysm diameter measurements for the follow-up of non-surgical patients were conducted using ultrasonography instead of the baseline CT scan, potentially introducing some variability due to differing techniques.

Supplementary Figures and Video:

Figure S1. Blood subtraction for SUV_{diff} correction

Figure S2. Anatomical slice location and segmentation of the aorta

Figure S3. RPI Calculation and representation

Figure S4. CCR2 radiotracer ([^{64}Cu] Cu-DOTA-ECL1i) PET/CT signal in AAA patients and non-AAA controls

Figure S5. CCR2 radiotracer ([^{64}Cu] Cu-DOTA-ECL1i) PET/CT signal in surgical, non-surgical AAA patients and non-AAA controls

Figure S6. PET/CT CCR2 signal intensity is associated with CCR2+ cell infiltration in surgical AAA specimens

Figure S7. PET/CT CCR2 signal intensity is associated with disease severity in surgical AAA specimens

Figure S8. CCR2 radiotracer ([^{64}Cu] Cu-DOTA-ECL1i) PET/CT signal detailed analysis in surgical and non-surgical AAA patients

Supplementary Tables:

Table S1. Demographics for AAA Surgical vs non-surgical AAAs

Table S2. Demographics for AAA High and Low CCR2 PET signal

Table S3. Summary of Mortality and AAA-related outcomes in AAA patients.

Figure S1. Blood subtraction for SUV_{diff} correction

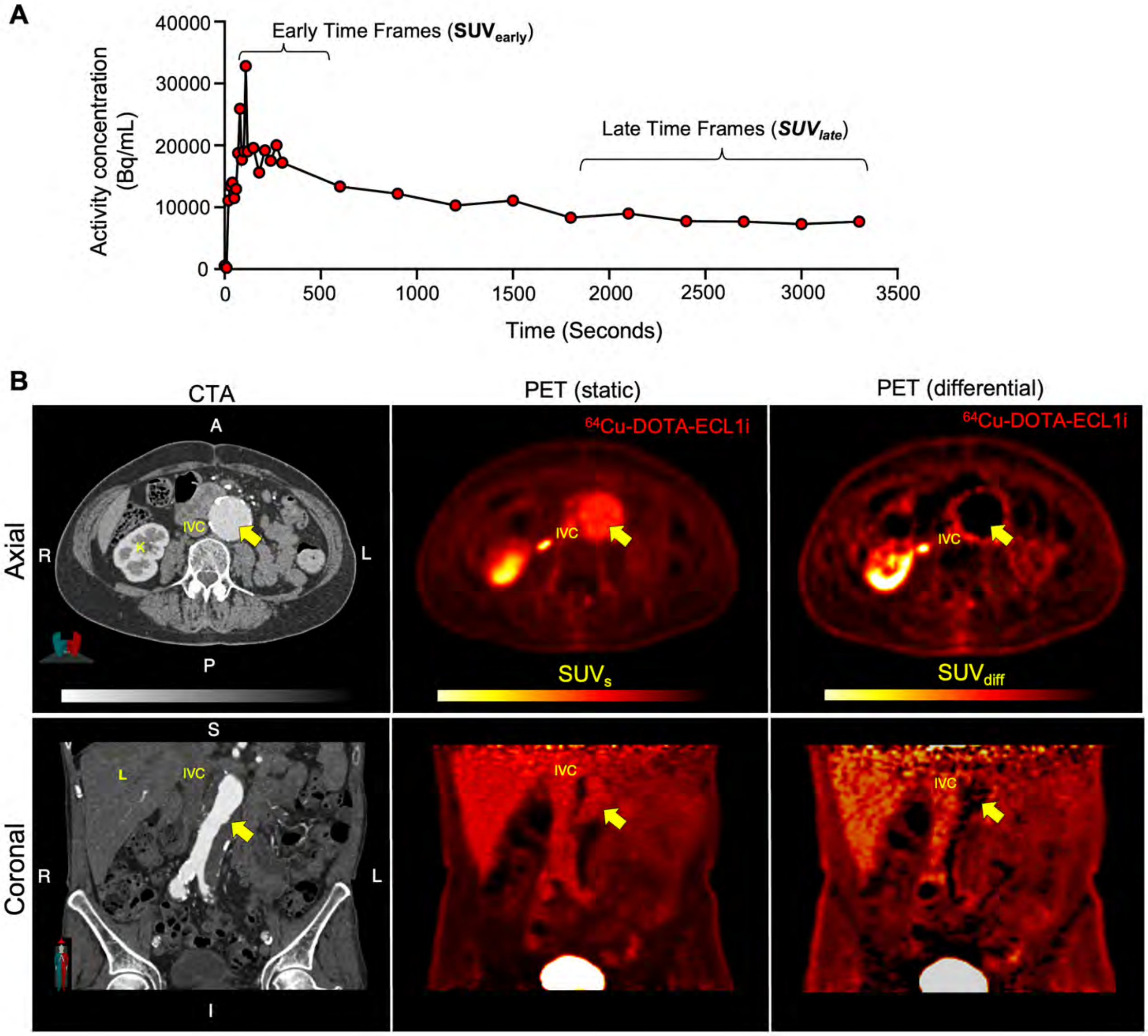

(**A**) Time activity curve and activity concentration of [^{64}Cu] Cu-DOTA-ECL1i throughout different time points measured at infrarenal segment of the IVC. (**B**) CTA of axial and coronal cuts representative of PET images. PET static represents the classic analysis of uptake from average frames (SUV_s). PET w/blood subtraction represents a novel analysis of uptake from the difference between early and late frames to correct the blood pool and spill over. Both CTA and PET/CT images correspond to the same patient. Kidney (K), Liver (L), Inferior Vena Cava (IVC): demonstrates the specific subtraction only to the abdominal aorta. The yellow arrow points at the Abdominal Aorta.

Figure S2. Anatomical slice location and segmentation of the aorta

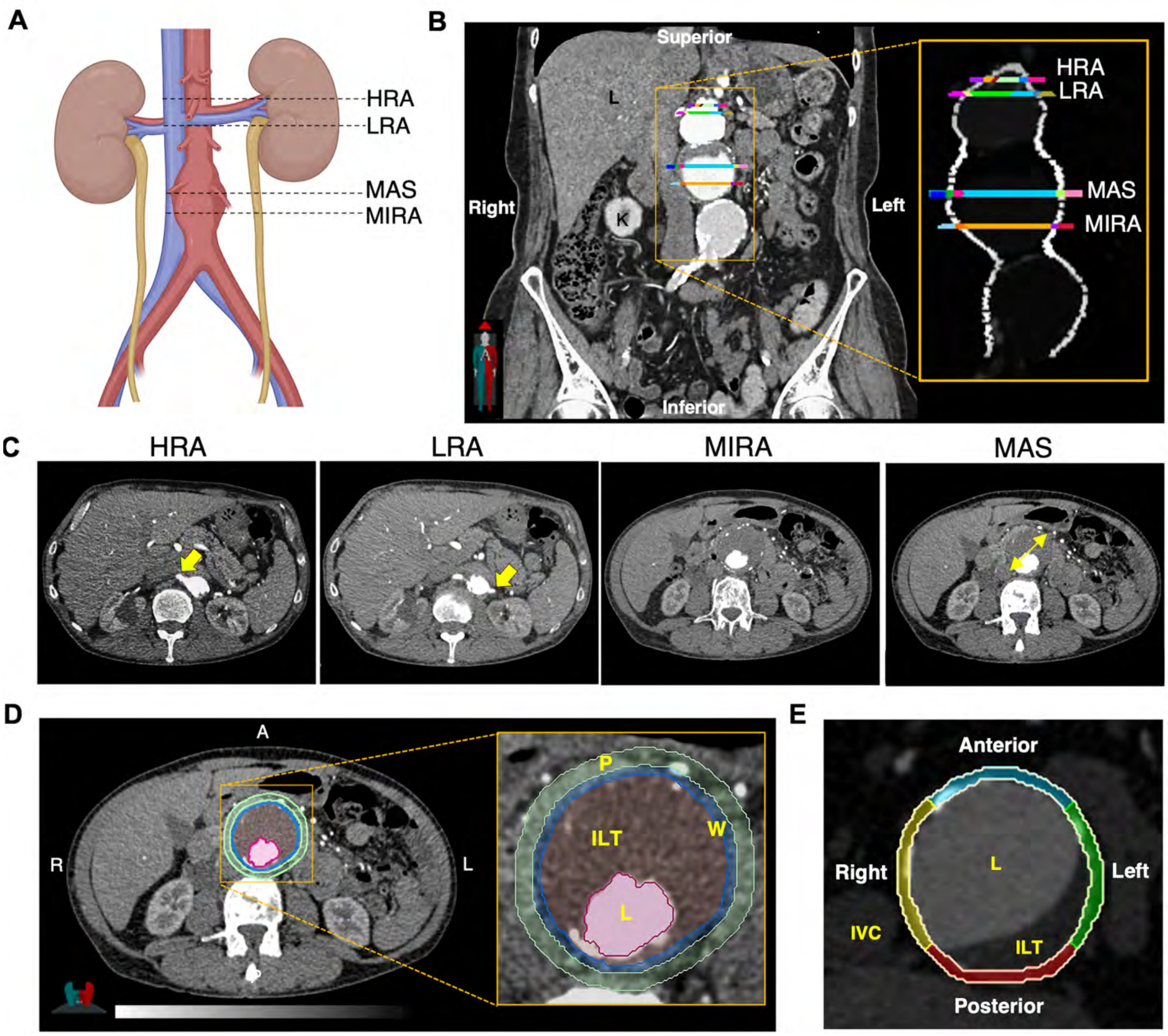

(**A** & **B**) Representative drawing and coronal CT cut of the defined anatomical slice locations. (**C**) Axial-cross CTA scan showing HRA, LRA, MIRA and MAS landmarks for defining the anatomical slice locations. (**D**) Segmentation (SOIs) of the AAA: P = Peri-aortic tissue (5mm thickness); W = Aortic Wall (2.5 mm thickness); ILT = Intraluminal thrombus; L = Aortic Lumen. (**E**) Quadrant sectoring of a cross-section to determine specific uptake within the wall and peri-aortic tissue. Figure was made using BioRender.com.

Figure S3. RPI Calculation and representation

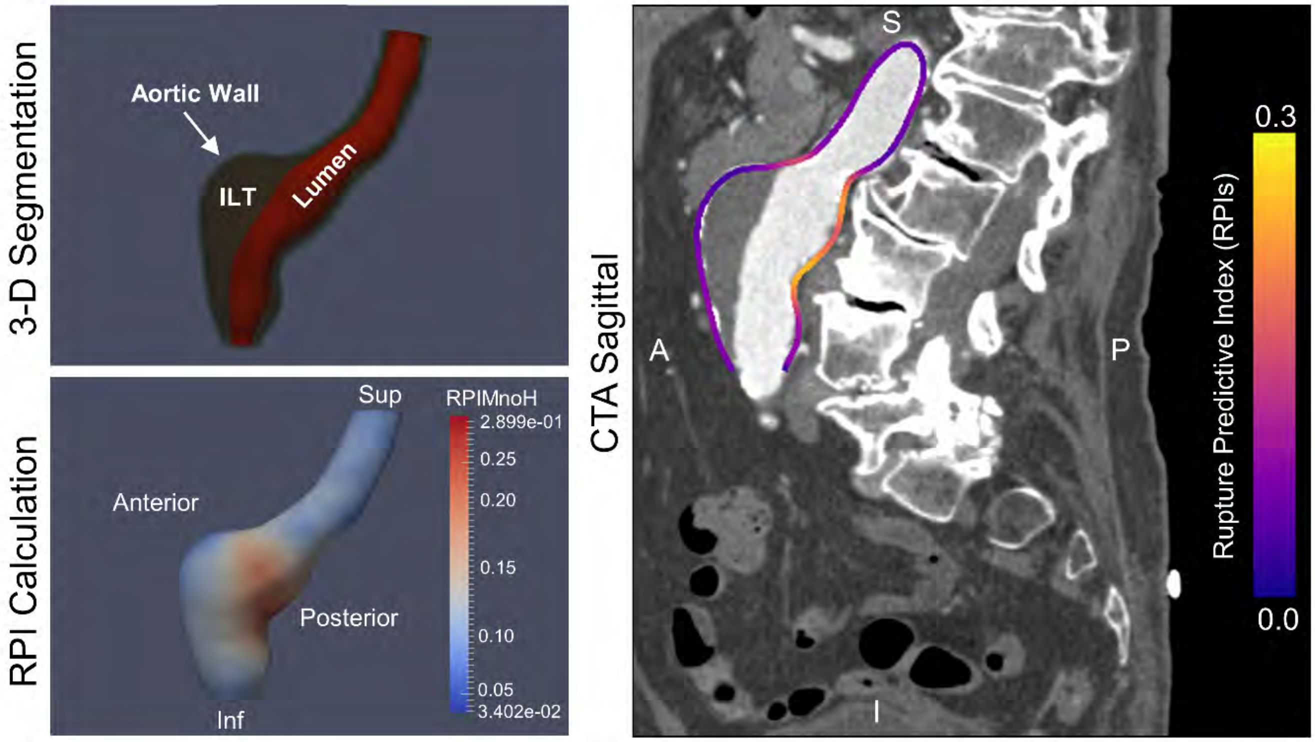

3-D Segmentation derived from subject's CTA aids in RPI calculation. RPI value is then transferred back to the patients CTA (sagittal view) to demonstrate the location of maximum wall stress within the abdominal aorta.

Figure S4. CCR2 radiotracer [^{64}Cu] Cu-DOTA-ECL1i PET/CT signal in AAA patients and non-AAA controls

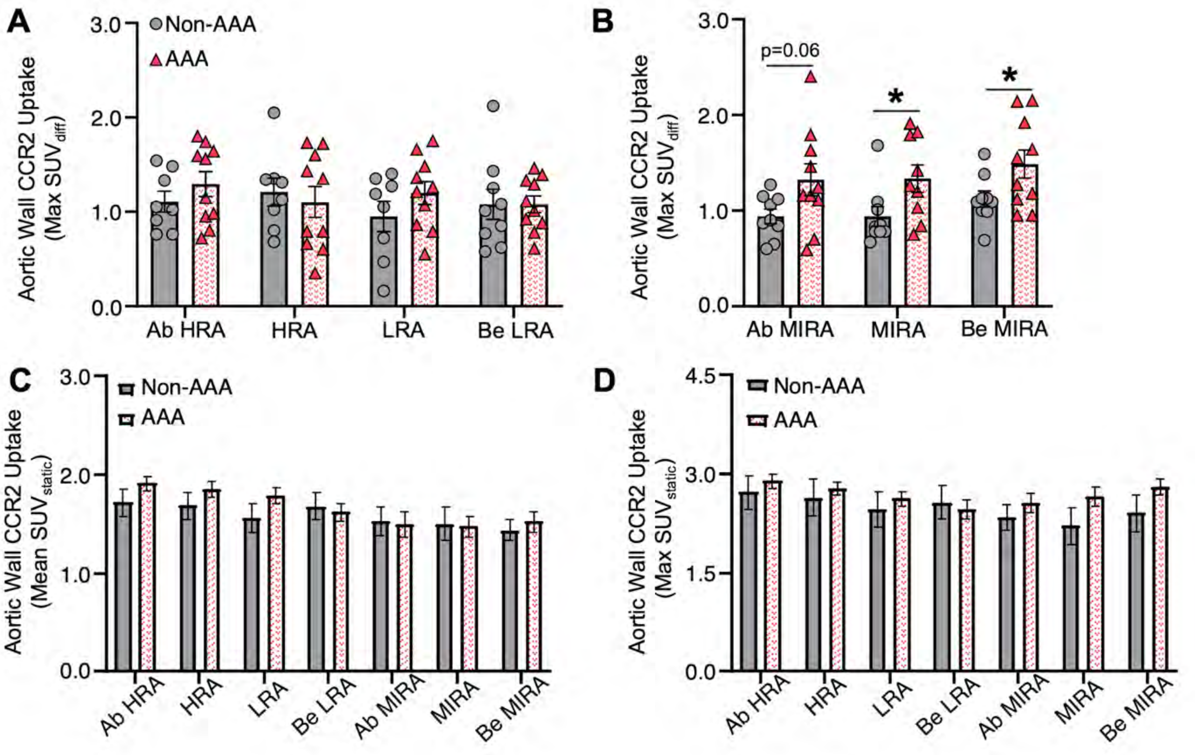

Max SUV_{diff} CCR2 radiotracer signal CCR2 along the peri-renal region aneurysm wall (**A**) and the MIRA region (**B**), in AAA and non-AAA groups. Wall CCR2 uptake using mean SUV_{static} (**C**) and max SUV_{static} (**D**) in different anatomical slice locations between AAA and non-AAA controls. Ab = Above. HRA = Highest Renal Artery. Be = Bellow. LRA = Lowest Renal Artery. MIRA = Mid infrarenal aorta.

Figure S5. CCR2 radiotracer ([^{64}Cu] Cu-DOTA-ECL1i) PET/CT signal in surgical, non-surgical AAA patients and non-AAA controls

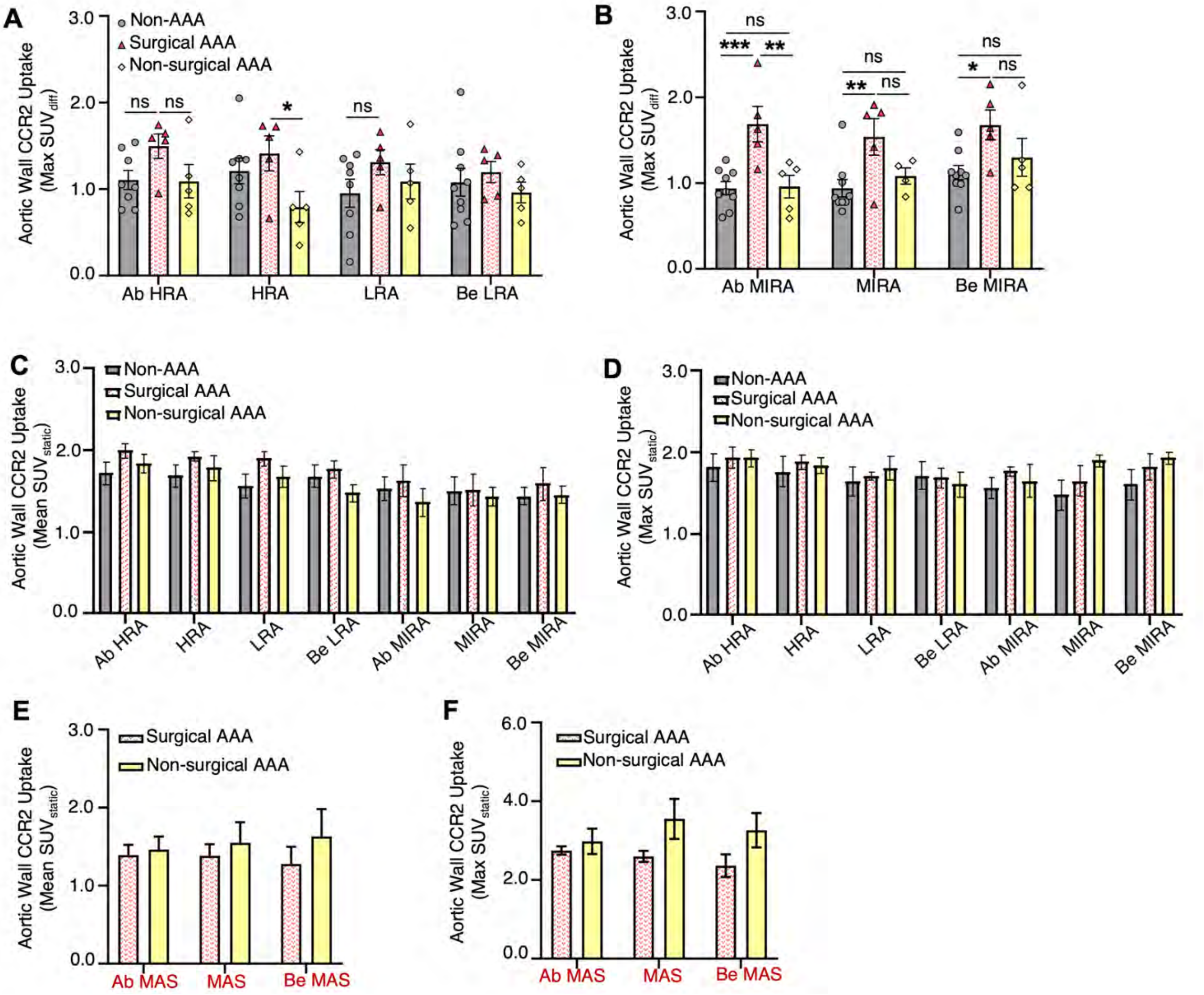

Aortic wall CCR2 signal using *max* SUV_{diff} along the peri-renal aorta (**A**) and the MIRA region (**B**). Wall CCR2 signal using *mean* SUV_{static} (**C**) and *max* SUV_{static} (**D**) in different anatomical slice locations among studied groups. Wall CCR2 signal using *mean* (**E**) and *max* (**F**) SUV_{diff} within the maximum aneurysm sac (MAS) between surgical and non-surgical AAA groups. Ab = Above, HRA = Highest Renal Artery, Be = Bellow LRA = Lowest Renal Artery and MIRA = Mid infrarenal aorta.

Figure S6. PET/CT CCR2 signal intensity is associated with CCR2+ cell infiltration in surgical AAA specimens

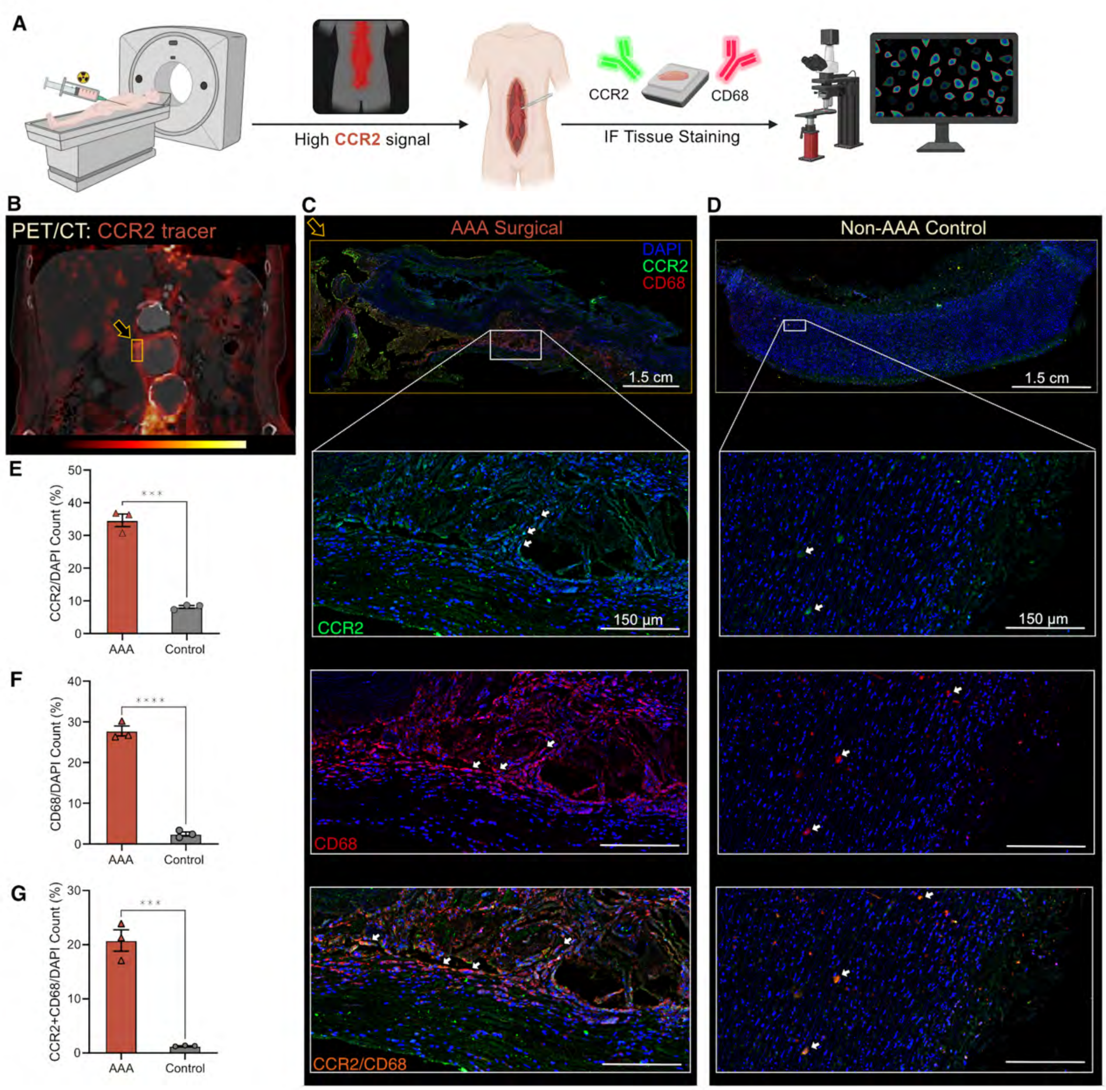

(**A**) Illustration demonstrating the different protocolar steps for the histopathological and IF analysis, figure was made using BioRender.com. (**B**) PET/CT CCR2 merged image demonstrating tracer intensity and location of surgical specimen collection. Immunofluorescent staining at 5x and 10x demonstrates CCR2 (green), CD68 (red) and DAPI (blue) positive cells in (**C**) AAA surgical specimens and (**D**) non-AAA controls. Quantification of the percentage of CCR2+ cells relative to DAPI (**E**), percentage of CD68+ cells relative to DAPI (**F**), and percentage of co-localized CCR2+ and CD68+ cells relative to DAPI (**G**).

Figure S7. PET/CT CCR2 signal intensity is associated with disease severity in surgical AAA specimens

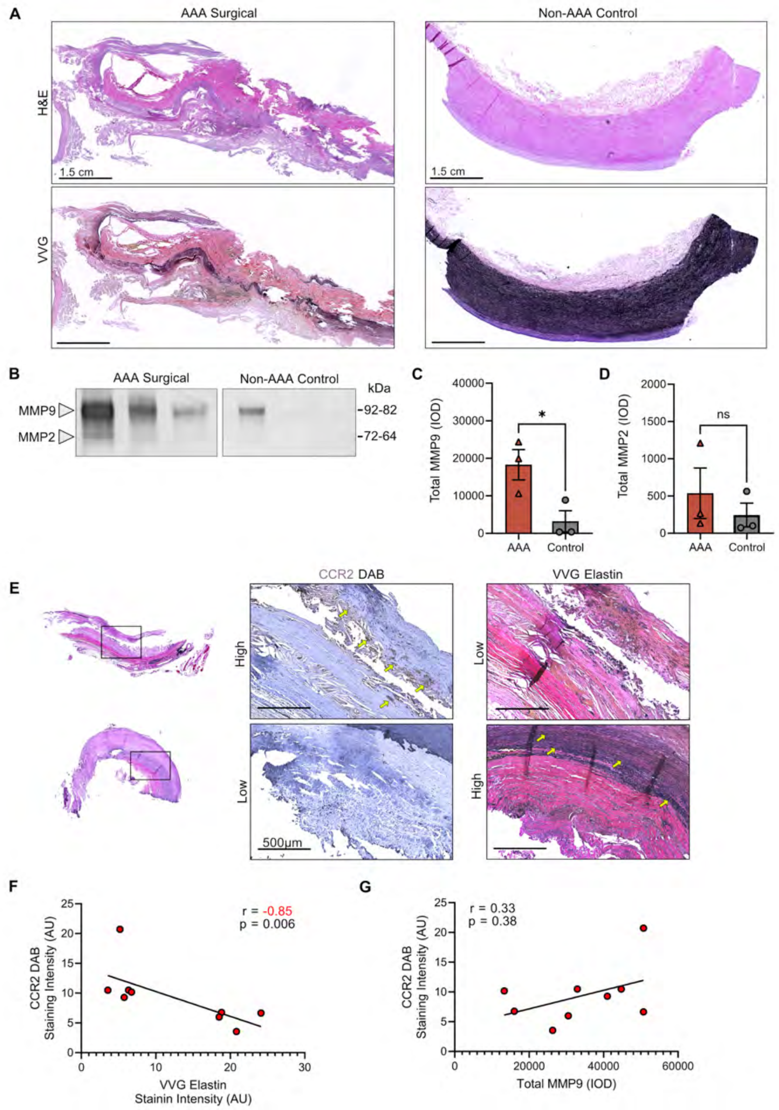

(**A**) Aortic tissue sample stained with VVG for elastin fibers (in black) at 10x demonstrated high breakdown in the AAA surgical when compared to non-AAA control. (**B**) Zymogram demonstrating total MMP9 and MMP2 measured by integrated optical density (IOD). (**C&D**) Total MMP9 and MMP2 activity levels demonstrates an increase in AAA surgical tissue, compared to non-AAA controls.

Figure S8. CCR2 radiotracer ([^{64}Cu] Cu-DOTA-ECL1i) PET/CT signal detailed analysis in surgical and non-surgical AAA patients

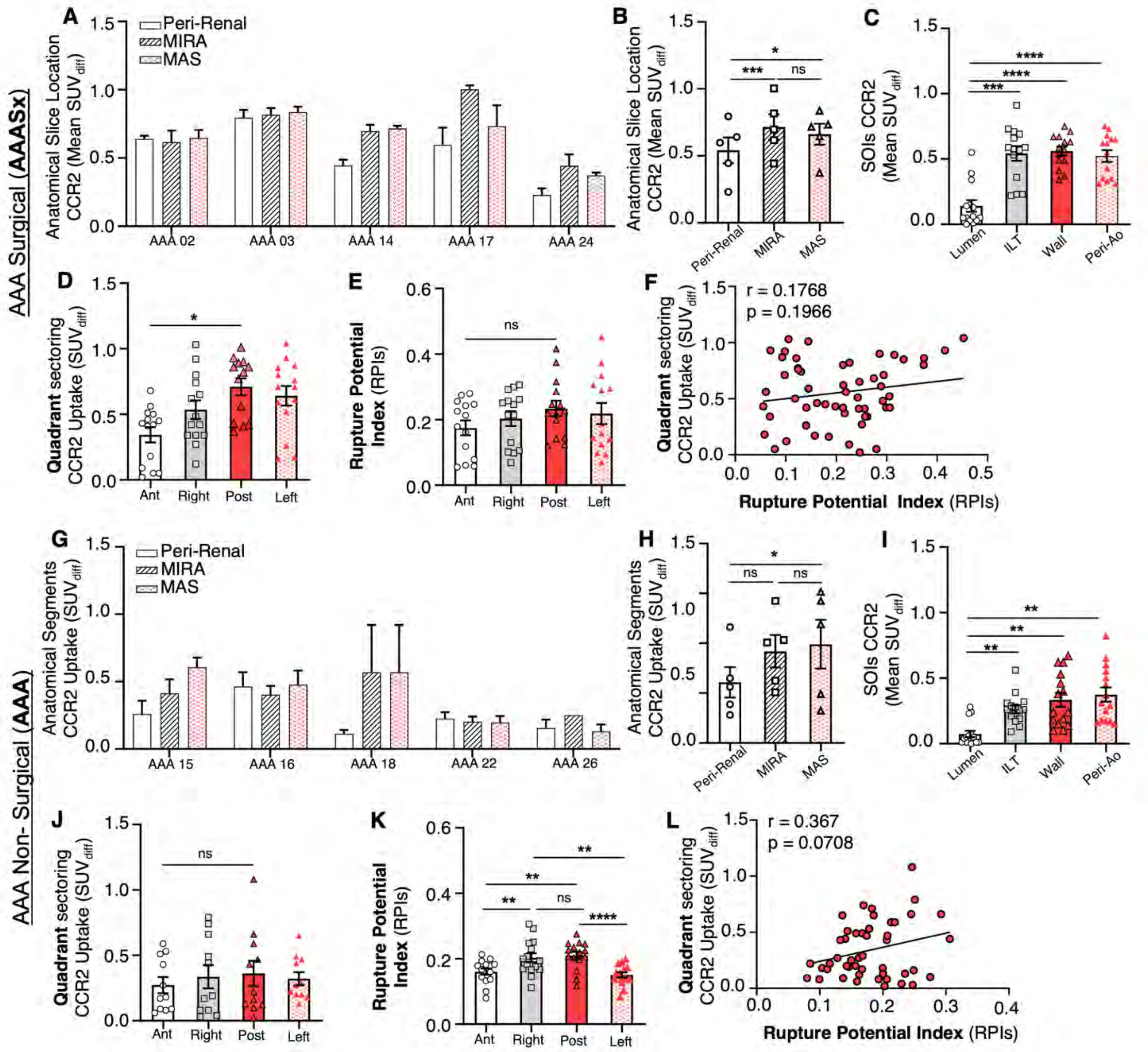

(**A&B**) Wall CCR2 signal intensity using SUV_{diff} at different anatomical slice locations, SOIs (**C**) and quadrants for surgical AAAs (**D**). (**E**) Mean RPI values for surgical AAA patients and (**F**) its direct correlation with CCR2 PET signal. (**G&H**) Wall CCR2 signal intensity using *mean* SUV_{diff} at different anatomical slice locations, SOIs (**I**) and quadrants for non-surgical AAAs (**J**). (**K**) Mean RPI values for non-surgical AAA patients and (**L**) its direct correlation with CCR2 signal. MAS = Maximum aneurysm sac. Ab = Above, HRA = Highest Renal Artery, Be = Bellow LRA = Lowest Renal Artery and MIRA = Mid infrarenal aorta. Ant = Anterior, Post = Posterior, ILT = Intraluminal Thrombus.

Table S1. Demographics for AAA Surgical vs non-surgical AAAs

		Non-Surgical AAA (N=5)	Surgical AAA (N=5)	P-value
**Age** mean (SD)		71 (8.1)	70.4 (7.4)	0.90
**Sex** (Male)		4 (80%)	3 (60%)	0.49
**Race**	White	5 (100%)	5 (100%)	N/A
	African American	0	0	
	Asian	0	0	
**Smoking Status**	Former	2 (40%)	2 (40%)	0.71
	Active	2 (40%)	1 (20%)	N/A
**GFR** mean (SD) mL/min		82 (14)	65.8 (17)	0.22
**Aneurysm Diameter** mean (SD)		4.3 (0.71)	5.4 (0.23)	**0.01**
**Concurrent Iliac Aneurysm**		2 (40%)	2 (40%)	1.00
**Co-morbidities**	Hypertension	4 (80%)	5 (100%)	0.29
	Diabetes Mellitus	0	0	N/A
	Hyperlipidemia	5 (100%)	5 (100%)	N/A
	Ischemic Heart Disease	3 (60%)	1 (20%)	0.19
	Peripheral Arterial Disease	0	1 (20%)	0.29
	Cerebrovascular Disease	2 (40.0%)	3 (60.0%)	0.52
**Medications**	Metformin	0	0	N/A
	Statin	4 (80.0%)	5 (100.0%)	0.29
	Beta blocker	1 (20.0%)	0	0.29
	CCB	3 (60.0%)	2 (40.0%)	0.52
	ACEI/ARB	3 (60.0%)	3 (60.0%)	1.00
	Antiplatelet	3 (60.0%)	3 (60.0%)	1.00
	Anticoagulant	2 (40.0%)	0	0.11
AAA =Abdominal Aortic Aneurysm; GFR = Glomerular Filtration Rate; CCB = Calcium Channel Blocker; ACEI = angiotensin-converting enzyme inhibitor; ARB = angiotensin receptor blocker; N/A = non-applicable; SD = Standard Deviation.				

AAA = Abdominal Aortic Aneurysm. GFR = Glomerular Filtration Rate. CCB = Calcium Channel Blocker. ACEI = Angiotensin converting enzyme inhibitors. ARB = Angiotensin receptor blocker. N/A = non-applicable. SD = Standard Deviation.

Table S2. Demographics for AAA High and Low CCR2 PET signal

		Low CCR2 (N=4)	High CCR2 (N=5)	P-value
**Age** mean (SD)		75.2 (9.9)	67.6 (3.2)	0.11
**Sex** (Male)		3 (75%)	4 (66.7%)	0.77
**Race**	White	4 (100%)	6 (100%)	N/A
	African American	0	0	
	Asian	0	0	
**Smoking Status**	Former	1 (25.0%)	3(50%)	0.51
	Active	1 (25.0%)	2 (53.3%)	N/A
**GFR** mean (SD) mL/min		86 (17)	67 (17)	0.16
**Aneurysm Diameter** mean (SD)		4.5 (0.4)	5.1 (0.8)	0.31
**Concurrent Iliac Aneurysm**		1 (25.0%)	3 (50%)	0.42
**Co-morbidities**	Hypertension	3 (75.0%)	6 (100%)	0.19
	Diabetes Mellitus	0	0	N/A
	Hyperlipidemia	4 (100%)	6 (100%)	N/A
	Ischemic Heart Disease	3 (75%)	1 (16.7%)	0.06
	Peripheral Arterial Disease	1 (25%)	0	0.19
	Cerebrovascular Disease	3 (75%)	2 (33.3%)	0.19
**Medications**	Metformin	0	0	N/A
	Statin	3 (75%)	6 (100%)	0.19
	Beta blocker	0	1 (16.7%)	0.38
	CCB	2 (50.0%)	3 (50.0%)	1.00
	ACEI/ARB	3 (75%)	3 (50.0%)	0.42
	Antiplatelet	3 (75.0%)	3 (50.0%)	0.42
	Anticoagulant	1 (25.0%)	1 (16.7%)	0.74

AAA =Abdominal Aortic Aneurysm; GFR = Glomerular Filtration Rate; CCB = Calcium Channel Blocker; ACEI = angiotensin-converting enzyme inhibitor; ARB = angiotensin receptor blocker; N/A = non-applicable; SD = Standard Deviation.

Table S3. Summary of Mortality and AAA-related outcomes in AAA patients.

Outcome		Surgical AAA (N=5)	Non-Surgical AAA (N=5)		
			Total	High CCR2 (N=2)	Low CCR2 (N=3)
**All-Cause Mortality**		0	0	0	0
**AAA-Related Events**	Composite Events	1	4	2	2
	Rupture	0	0	0	0
	Repair	0	2	1	1
	Endoleak	1	2	1	1
**Mean AAA Growth (mm)**	6 Months	N/A	0.7	1.9	0.3
	1 Year	N/A	5	10	1

AAA = Abdominal Aortic Aneurysm; N/A = non-applicable.

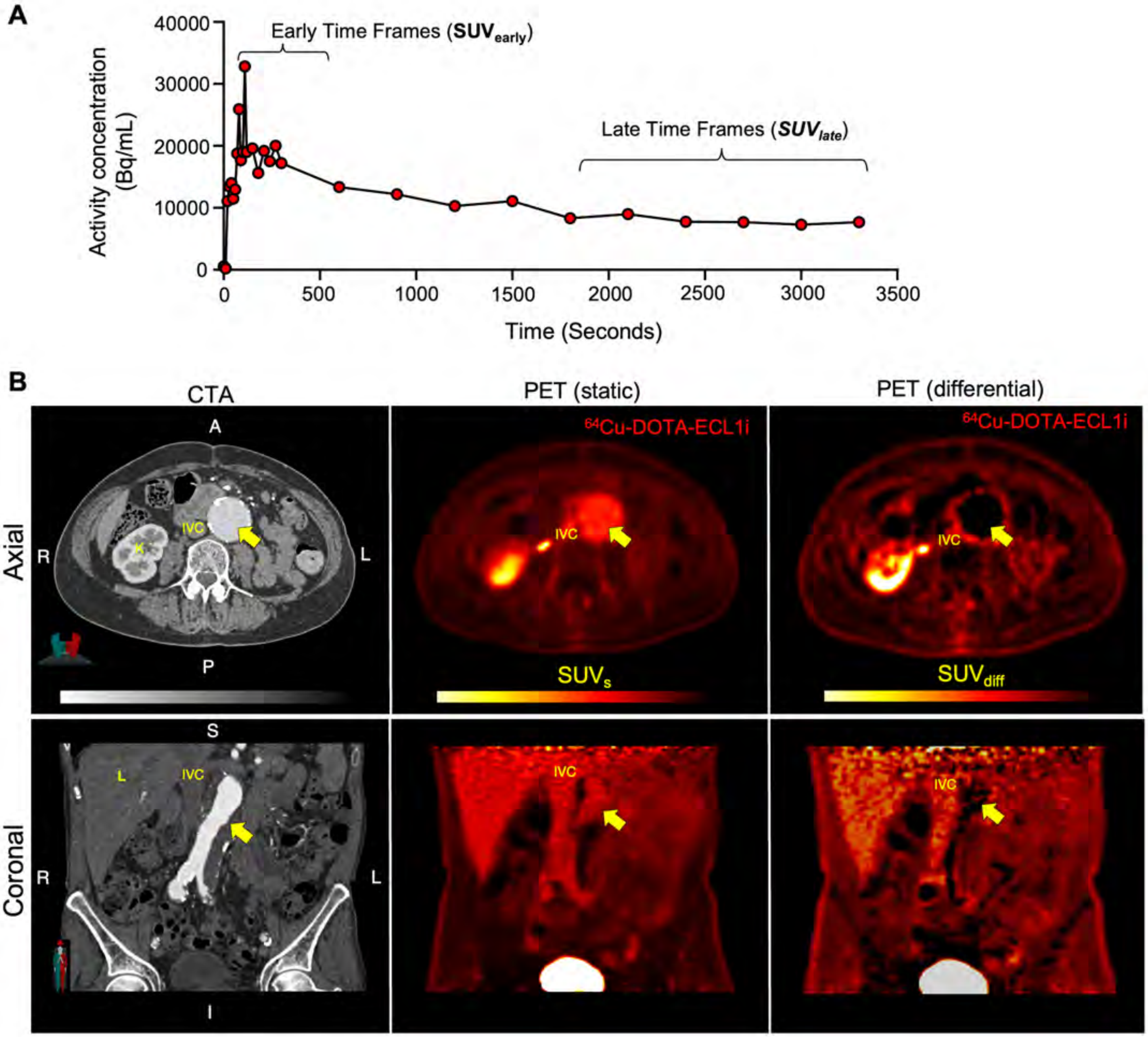
A
Early Time Frames (SUVearly)
Late Time Frames (SUVlate)
Activity concentration (Bq/mL)
40000
30000
20000
10000
0
0
500
1000
1500
2000
2500
3000
3500
Time (Seconds)
B
CTA
PET (static)
PET (differential)
64Cu-DOTA-ECL1i
64Cu-DOTA-ECL1i
Axial
A
R
L
P
K
IVC
IVC
IVC
SUVs
SUVdiff
Coronal
S
I
L
IVC
IVC
IVC

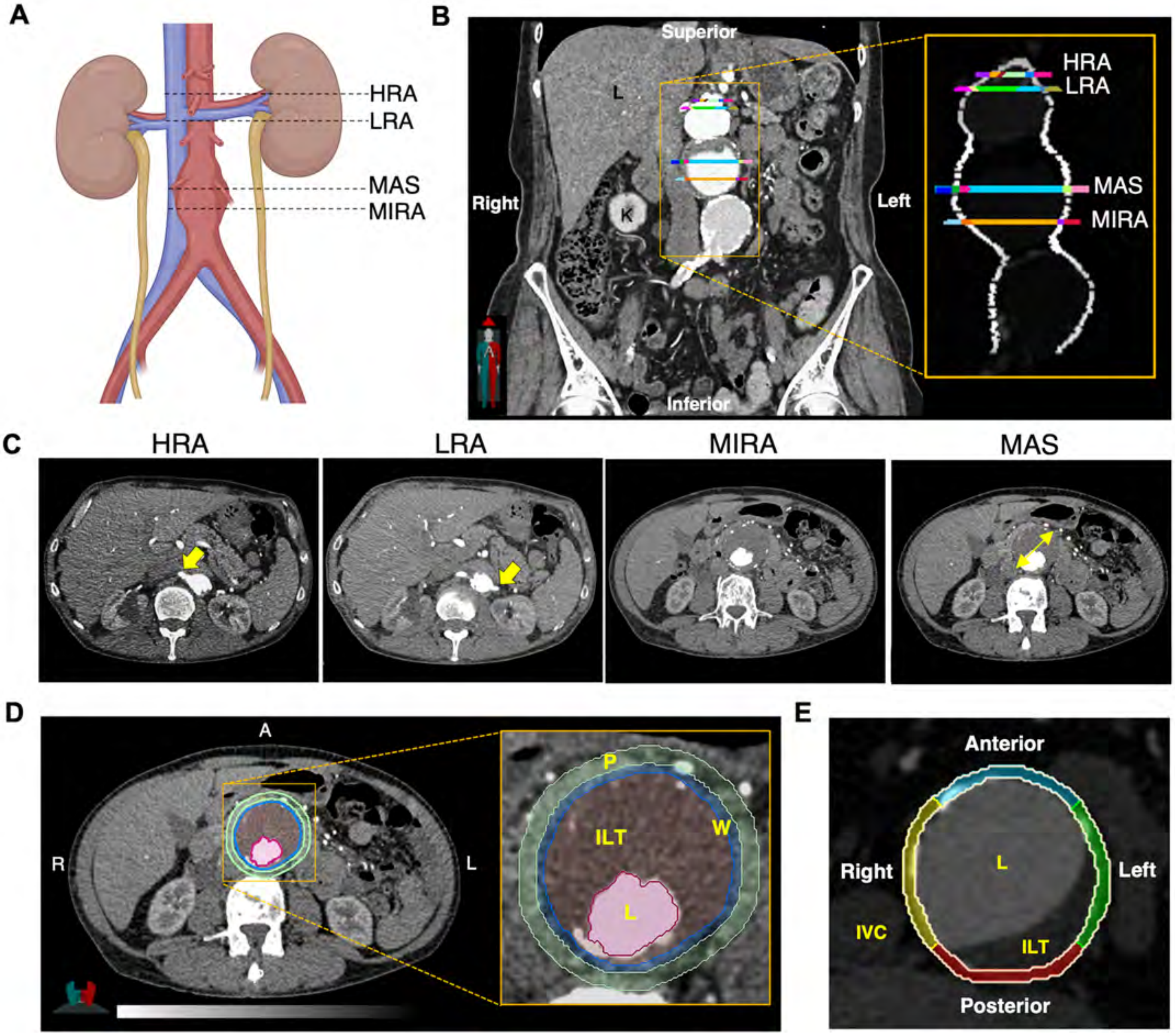
A
HRA
LRA
MAS
MIRA
B
Superior
L
Right
K
Left
Inferior
HRA
LRA
MAS
MIRA
C
HRA
LRA
MIRA
MAS
D
A
R
L
P
ILT
W
L
E
Anterior
Right
L
Left
IVC
ILT
Posterior

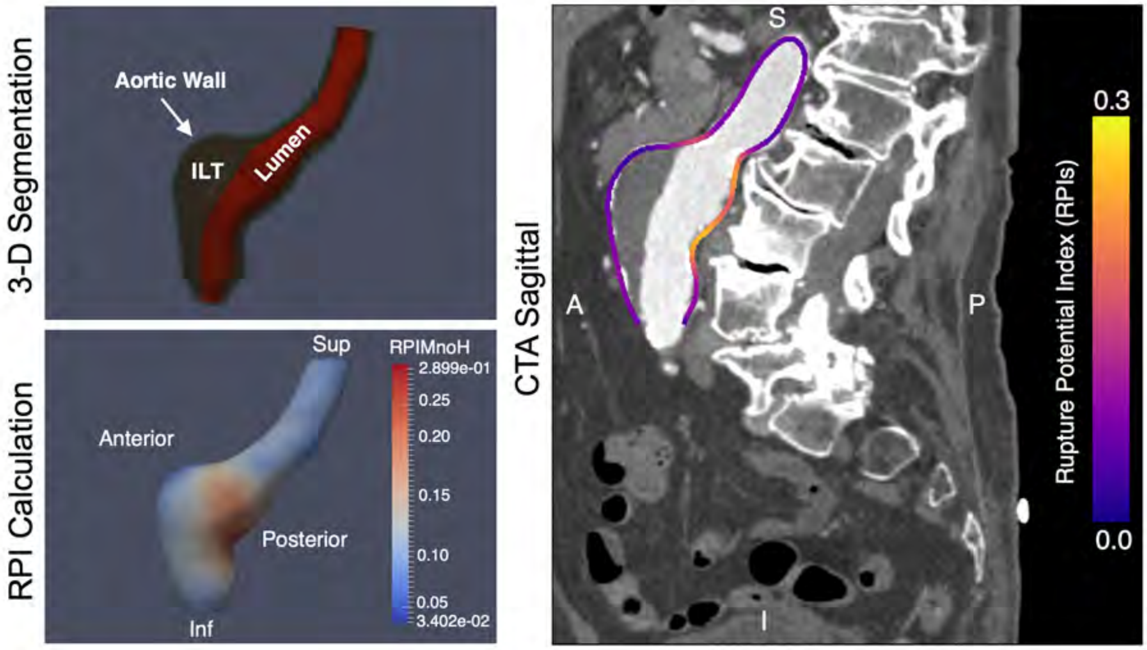

3-D Segmentation
Aortic Wall
ILT
Lumen
RPI Calculation
Sup
Anterior
Posterior
Inf
RPIMnoH
2.899e-01
0.25
0.20
0.15
0.10
0.05
3.402e-02
CTA Sagittal
S
A
P
I
0.3
Rupture Potential Index (RPIs)
0.0

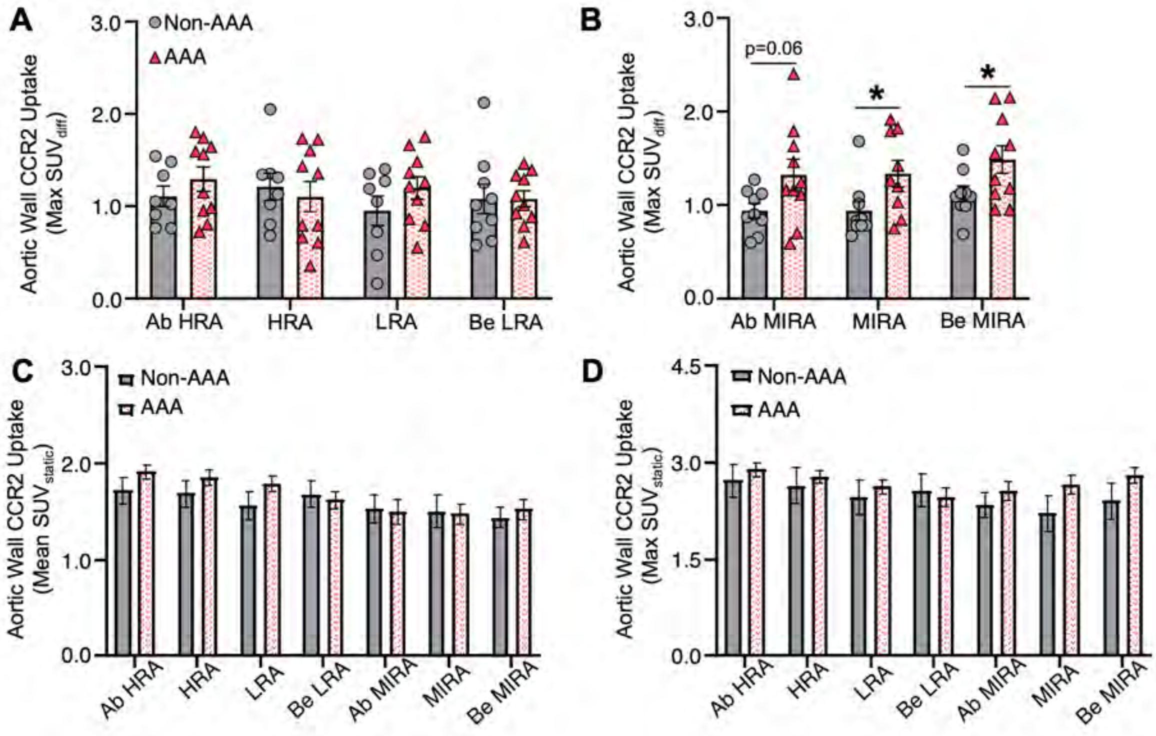
A
B
C
D
Non-AAA
AAA
Aortic Wall CCR2 Uptake (Max SUV_diff)
Aortic Wall CCR2 Uptake (Mean SUV_static)
Aortic Wall CCR2 Uptake (Max SUV_static)
p=0.06
*
Ab HRA
HRA
LRA
Be LRA
Ab MIRA
MIRA
Be MIRA

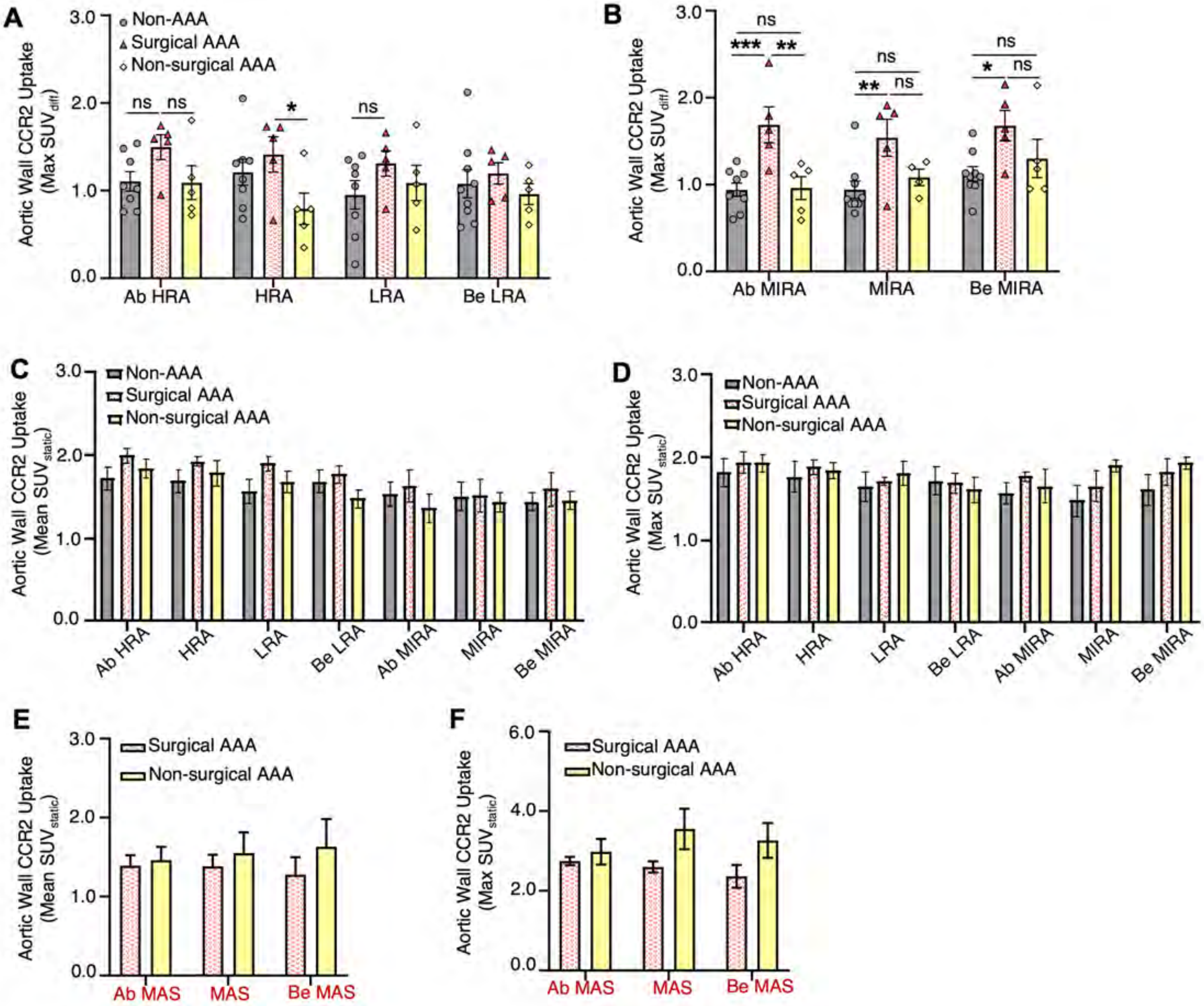
A
B
C
D
E
F
Aortic Wall CCR2 Uptake (Max SUV$_{diff}$)
Aortic Wall CCR2 Uptake (Mean SUV$_{static}$)
Aortic Wall CCR2 Uptake (Max SUV$_{static}$)
Non-AAA
Surgical AAA
Non-surgical AAA
Ab HRA
HRA
LRA
Be LRA
Ab MIRA
MIRA
Be MIRA
Ab MAS
MAS
Be MAS
ns
*
**
***

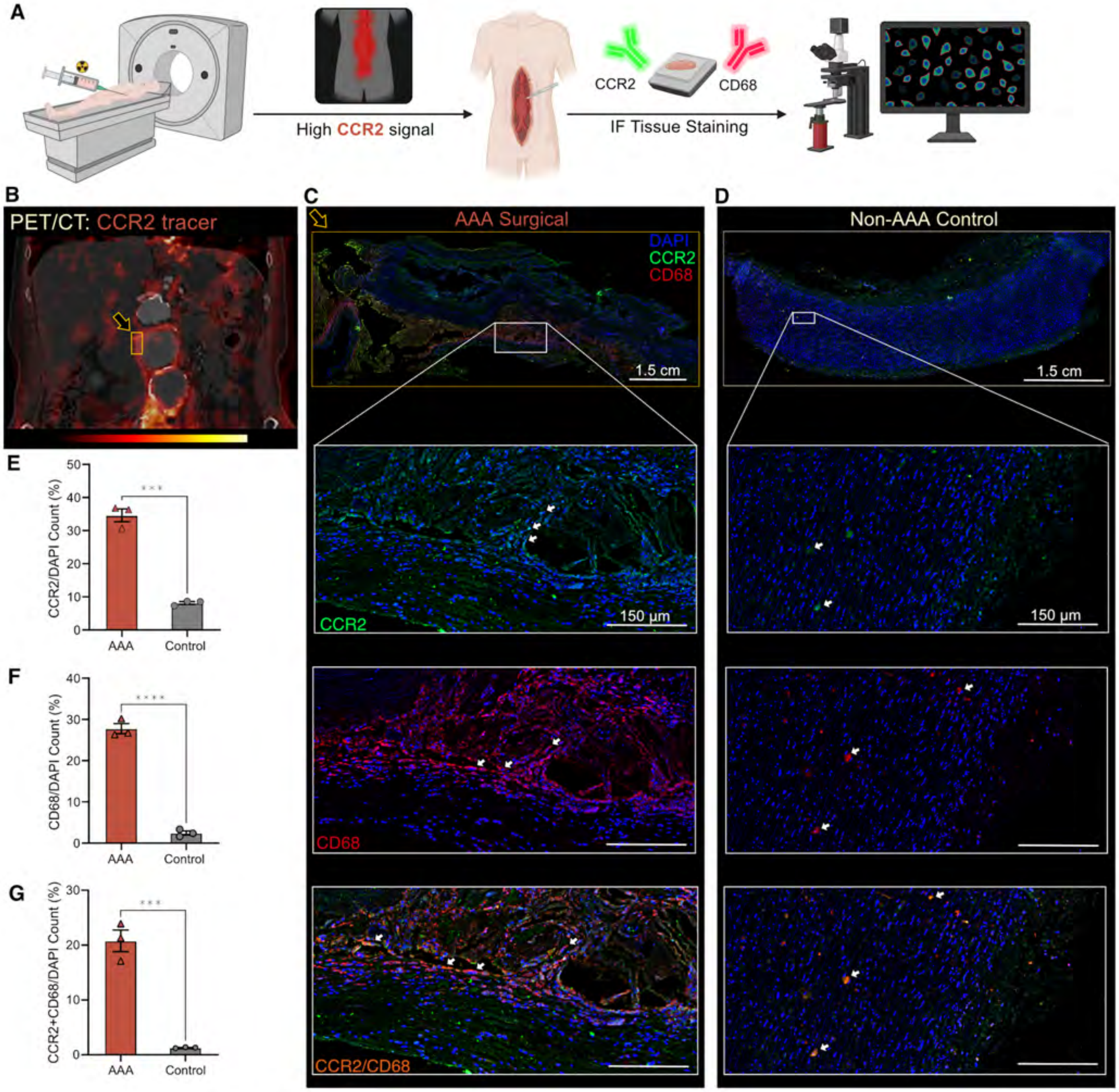
A
High CCR2 signal
CCR2
CD68
IF Tissue Staining
B
PET/CT: CCR2 tracer
C
AAA Surgical
DAPI
CCR2
CD68
1.5 cm
150 µm
CCR2
CD68
CCR2/CD68
D
Non-AAA Control
1.5 cm
150 µm
E
CCR2/DAPI Count (%)
AAA
Control
F
CD68/DAPI Count (%)
AAA
Control
G
CCR2+CD68/DAPI Count (%)
AAA
Control

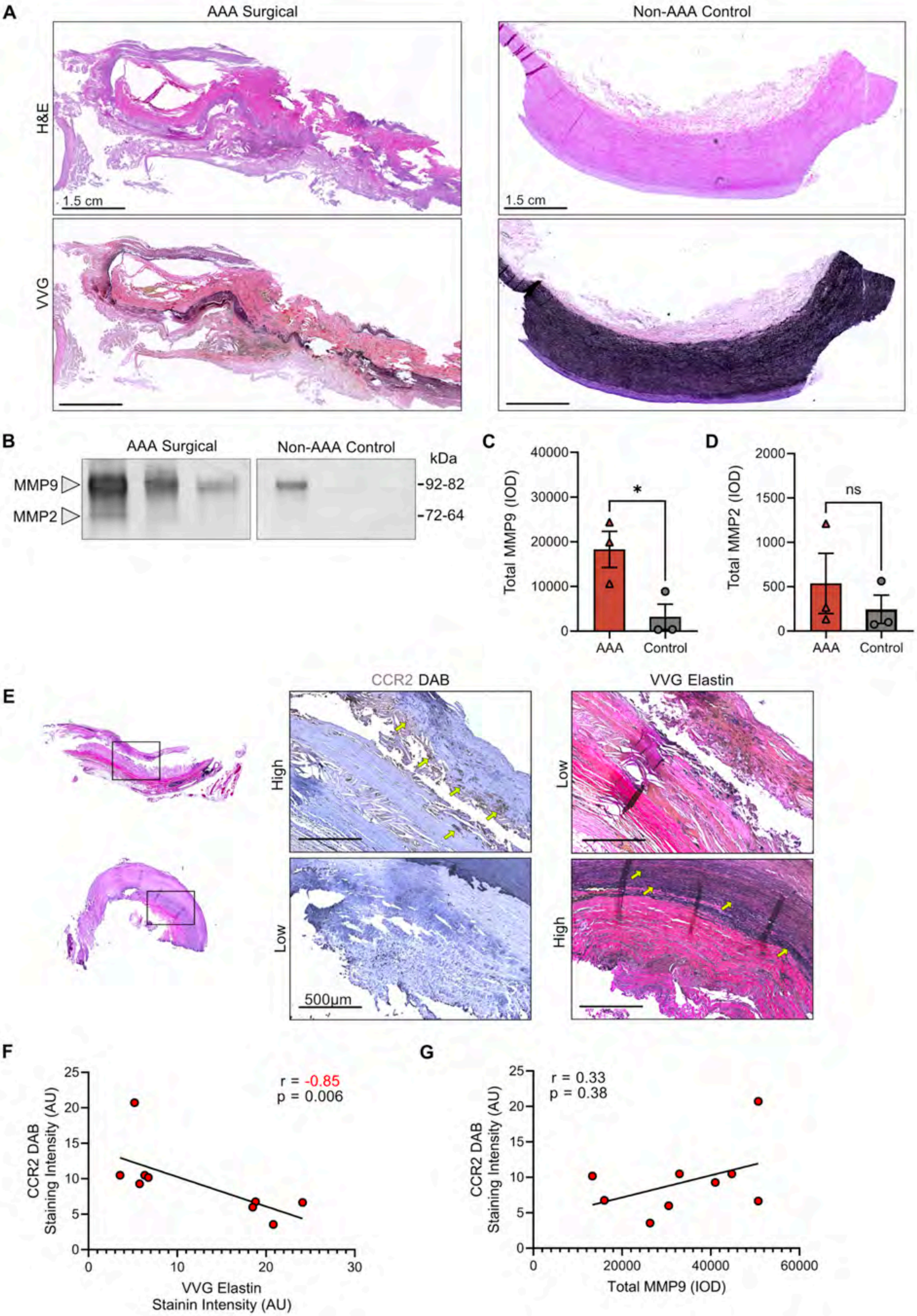
A
AAA Surgical
Non-AAA Control
H&E
VVG
1.5 cm
1.5 cm
B
AAA Surgical
Non-AAA Control
kDa
MMP9
MMP2
92-82
72-64
C
Total MMP9 (IOD)
40000
30000
20000
10000
0
*
AAA
Control
D
Total MMP2 (IOD)
2000
1500
1000
500
0
ns
AAA
Control
E
CCR2 DAB
VVG Elastin
High
Low
Low
High
500µm
F
r = -0.85
p = 0.006
CCR2 DAB
Staining Intensity (AU)
VVG Elastin
Stainin Intensity (AU)
G
r = 0.33
p = 0.38
CCR2 DAB
Staining Intensity (AU)
Total MMP9 (IOD)

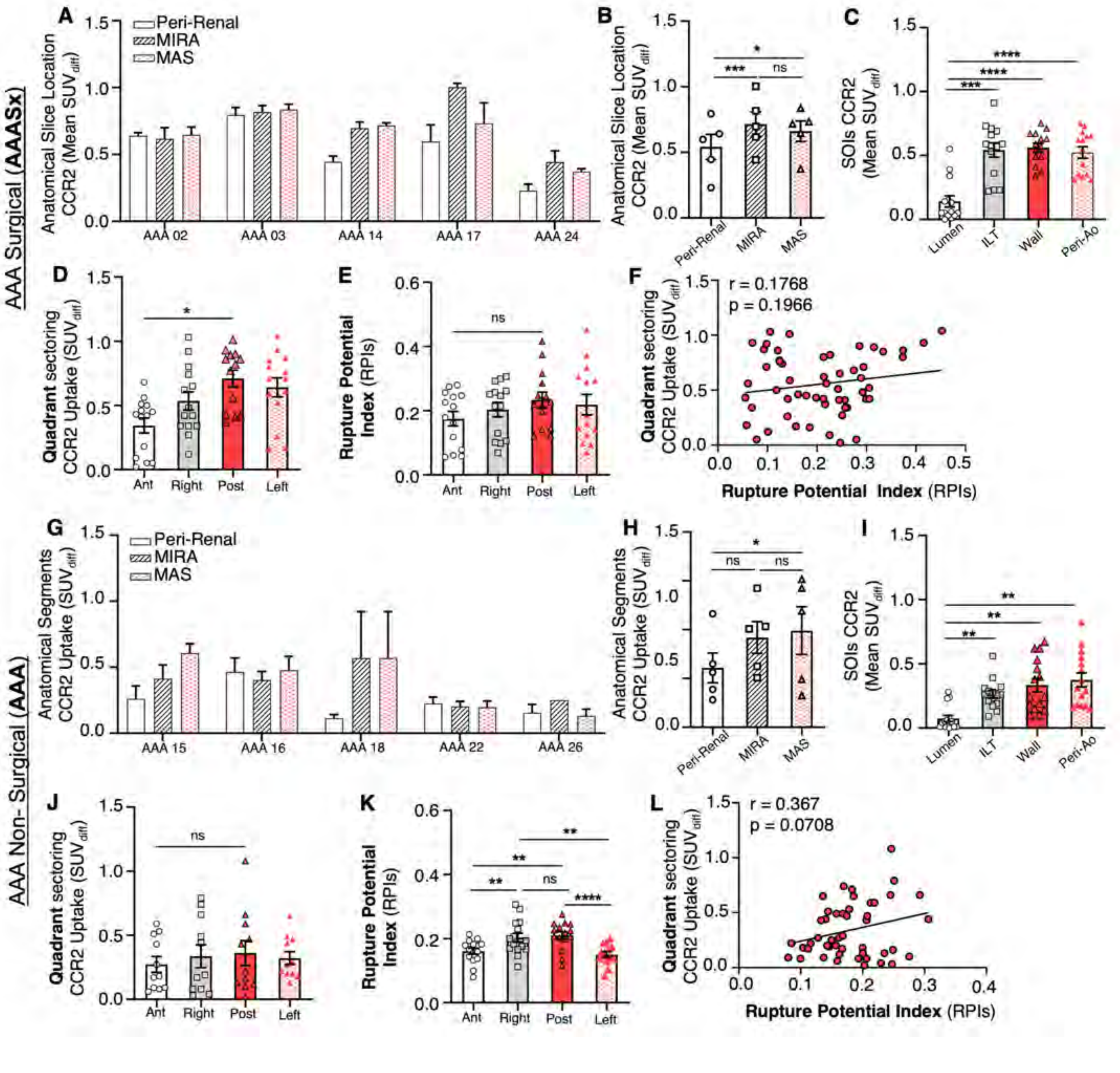
AAA Surgical (AAASx)
AAA Non- Surgical (AAA)
A
Peri-Renal
MIRA
MAS
Anatomical Slice Location CCR2 (Mean SUV$_{diff}$)
AAA 02
AAA 03
AAA 14
AAA 17
AAA 24
B
Anatomical Slice Location CCR2 (Mean SUV$_{diff}$)
Peri-Renal
MIRA
MAS
C
SOIs CCR2 (Mean SUV$_{diff}$)
Lumen
ILT
Wall
Peri-Ao
D
Quadrant sectoring CCR2 Uptake (SUV$_{diff}$)
Ant
Right
Post
Left
E
Rupture Potential Index (RPIs)
F
r = 0.1768
p = 0.1966
Quadrant sectoring CCR2 Uptake (SUV$_{diff}$)
Rupture Potential Index (RPIs)
G
Peri-Renal
MIRA
MAS
Anatomical Segments CCR2 Uptake (SUV$_{diff}$)
AAA 15
AAA 16
AAA 18
AAA 22
AAA 26
H
Anatomical Segments CCR2 Uptake (SUV$_{diff}$)
I
SOIs CCR2 (Mean SUV$_{diff}$)
J
Quadrant sectoring CCR2 Uptake (SUV$_{diff}$)
K
Rupture Potential Index (RPIs)
L
r = 0.367
p = 0.0708
Quadrant sectoring CCR2 Uptake (SUV$_{diff}$)
Rupture Potential Index (RPIs)

49 Spadina Ave. Suite 200
Toronto ON M5V 2J1 Canada
www.biorender.com

Confirmation of Publication and Licensing Rights - Open Access

March 16th, 2025

Subscription Type:	*Institution - Academic*
Agreement number:	*VZ2816RF20*
Publisher Name:	*Theranostics*
Figure Title:	*Graphical abstract*
Citation to Use:	*Created in BioRender. Arif, B. (2025) https://BioRender.com/h09h668*

To whom this may concern,

This document ("Confirmation") hereby confirms that Science Suite Inc. dba BioRender ("BioRender") has granted the following BioRender user: Batool Arif ("User") a BioRender Academic Publication License in accordance with BioRender's Terms of Service and Academic License Terms ("License Terms") to permit such User to do the following on the condition that all requirements in this Confirmation are met:

1) publish their Completed Graphics created in the BioRender Services containing both User Content and BioRender Content (as both are defined in the License Terms) in publications (journals, textbooks, websites, etc.); and
2) sublicense such Completed Graphics under "open access" publication sublicensing models such as CC-BY 4.0 and more restrictive models, so long as the conditions set forth herein are fully met.

Requirements of User:

1) All Completed Graphics to be published in any publication (journals, textbooks, websites, etc.) must be accompanied by the following citation either as a caption, footnote or reference for each figure that includes a Completed Graphic:
"Created in BioRender. Arif, B. (2025) https://BioRender.com/h09h668 ".
2) All terms of the License Terms including all Prohibited Uses are fully complied with. E.g. For Academic License Users, no commercial uses (beyond publication in journals, textbooks or websites) are permitted without obtaining or switching to a BioRender Industry Plan.
3) A Reader (defined below) may request that the User allow their figure to be a public template for Readers to view, copy, and modify the figure. It is up to the User to determine what level of access to grant.

Open-Access Journal Readers:

Open-Access journal readers ("Reader") who wish to view and/or re-use a particular Completed Graphic in an Open-Access journal subject to CC-BY sublicensing may do so by clicking on the URL link in the applicable citation for the subject Completed Graphic.

The re-use/modification options below are available after the Reader requests the User to adapt their

figure as a BioRender template and the User has granted such access.

1) View-Only/Free Plan Use: A Reader who wishes to only view the Completed Graphic may do so in the BioRender Services as either a BioRender Free Plan user or simply as a viewer. By becoming a BioRender Free Plan user, the Reader may view, modify and re-use the Completed Graphic as permitted under BioRender's Basic License Terms (e.g. personal use only, no publishing or commercial use permitted).
2) Re-Use/Publish with No Modifications: For any re-use and re-publication of a Completed Graphic with no modification(s) to the Completed Graphic made by the Reader, a Reader may do so by citing the original author using the citation noted above with the Completed Graphic. The Reader must also comply with the underlying License Terms which apply to the Completed Graphic as noted above (e.g. no commercial use for Academic License).
3) Re-Use/Publish with Modifications: For any re-use and re-publication of a Completed Graphic with a modification(s) made by the Reader, the Reader may do so by becoming a BioRender user themselves under either an Academic or Industry Plan, citing the original author using the citation noted above with the Completed Graphic and complying with the applicable License Terms.

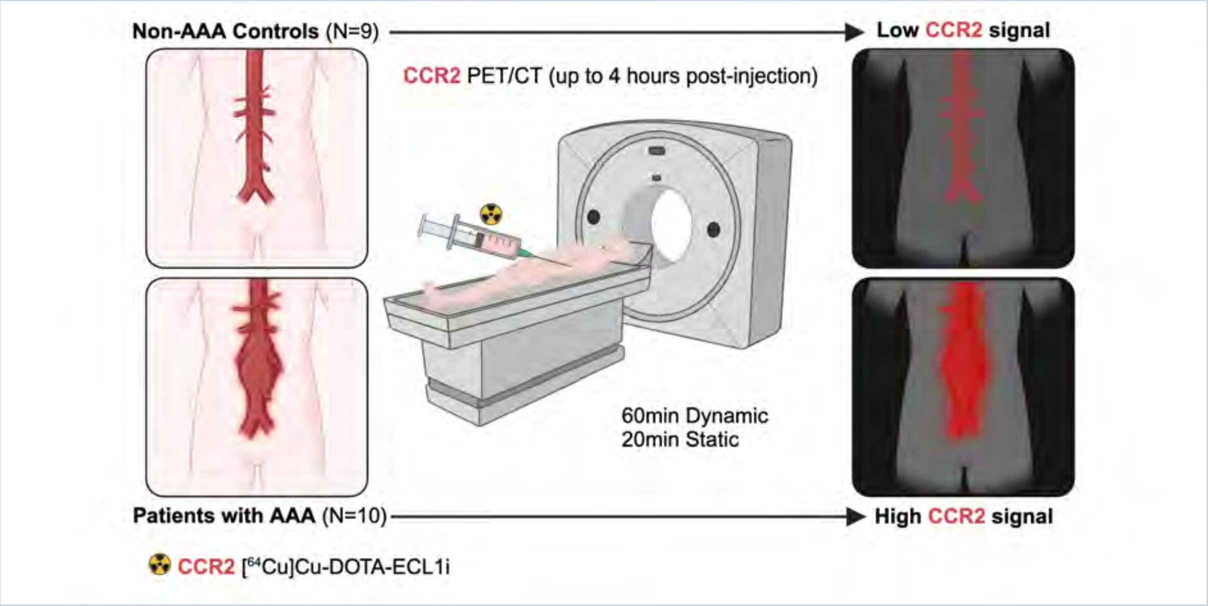

For any questions regarding this document, or other questions about publishing with BioRender, please refer to our BioRender Publication Guide, or contact BioRender Support at support@biorender.com.
